# G-CSF does not influence C2C12 myogenesis despite receptor expression in healthy and dystrophic skeletal muscle

**DOI:** 10.3389/fphys.2014.00170

**Published:** 2014-05-01

**Authors:** Craig R. Wright, Erin L. Brown, Paul A. Della-Gatta, Alister C. Ward, Gordon S. Lynch, Aaron P. Russell

**Affiliations:** ^1^Centre for Physical Activity and Nutrition, School of Exercise and Nutrition Sciences, Deakin UniversityBurwood, VIC, Australia; ^2^Molecular and Medical Research SRC, School of Medicine, Deakin UniversityWaurn Ponds, VIC, Australia; ^3^Basic and Clinical Myology Laboratory, Department of Physiology, The University of MelbourneVIC, Australia

**Keywords:** G-CSF, cytokine receptor, skeletal muscle, duchenne muscular dystrophy, *mdx*, C2C12, proliferation, differentiation

## Abstract

Granulocyte-colony stimulating factor (G-CSF) increases recovery of rodent skeletal muscles after injury, and increases muscle function in rodent models of neuromuscular disease. However, the mechanisms by which G-CSF mediates these effects are poorly understood. G-CSF acts by binding to the membrane spanning G-CSFR and activating multiple intracellular signaling pathways. Expression of the G-CSFR within the haematopoietic system is well known, but more recently it has been demonstrated to be expressed in other tissues. However, comprehensive characterization of G-CSFR expression in healthy and diseased skeletal muscle, imperative before implementing G-CSF as a therapeutic agent for skeletal muscle conditions, has been lacking. Here we show that the G-CSFR is expressed in proliferating C2C12 myoblasts, differentiated C2C12 myotubes, human primary skeletal muscle cell cultures and in mouse and human skeletal muscle. In *mdx* mice, a model of human Duchenne muscular dystrophy (DMD), G-CSF mRNA and protein was down-regulated in limb and diaphragm muscle, but circulating G-CSF ligand levels were elevated. G-CSFR mRNA in the muscles of *mdx* mice was up-regulated however steady-state levels of the protein were down-regulated. We show that G-CSF does not influence C_2_C_12_ myoblast proliferation, differentiation or phosphorylation of Akt, STAT3, and Erk1/2. Media change alone was sufficient to elicit increases in Akt, STAT3, and Erk1/2 phosphorylation in C_2_C_12_ muscle cells and suggest previous observations showing a G-CSF increase in phosphoprotein signaling be viewed with caution. These results suggest that the actions of G-CSF may require the interaction with other cytokines and growth factors *in vivo*, however these data provides preliminary evidence supporting the investigation of G-CSF for the management of muscular dystrophy.

## Introduction

Skeletal muscle atrophy is a devastating condition that is characteristic of neuromuscular diseases such as Duchenne muscular dystrophy (DMD) and many chronic diseases including cancer, heart disease, chronic obstructive pulmonary disease (COPD), sepsis and AIDS (Jagoe and Goldberg, 2001). As a result, skeletal muscle atrophy is a strong predictor of morbidity and mortality associated with cardiovascular, musculoskeletal, nervous, renal, and respiratory diseases, as well as cancer, which are among the leading causes of death worldwide (Murray and Lopez, 1997; Dal-Re, 2011). Therefore, development of therapeutic strategies to reduce muscle wasting while enhancing muscle regeneration and growth is required to improve clinical outcomes.

Granulocyte-colony stimulating factor (G-CSF, encoded by *CSF3*) is a cytokine that stimulates haematopoietic stem cell mobilization, myelopoiesis, and particularly the production and activation of neutrophils (Roberts, 2005). G-CSF exerts its effects by binding to the G-CSF receptor (G-CSFR, encoded by *CSF3R*) on target cells. G-CSFR is highly expressed on haematopoietic stem cells and various myeloid cells (Nagata and Fukunaga, 1991), but also on other cell types including neural tissue (Yata et al., 2007; Pitzer et al., 2008, 2010), liver stem cells (Piscaglia et al., 2007) and cardiac muscle (Li et al., 2006, 2007; Takano et al., 2006; Ueda et al., 2006; Shimoji et al., 2010). These observations suggest that G-CSF may regulate cells outside of the haematopoietic system.

Skeletal muscle precursor cells, retinal muscle, and muscles of the tongue have been shown to express G-CSFR during embryonic development (Kirsch et al., 2008; Oishi et al., 2008). More recently, G-CSFR expression has been also reported on proliferating C2C12 myoblasts using immunofluorescence and Western blotting (Hara et al., 2011). However, mRNA and protein levels of G-CSF and the G-CSFR have not been investigated in differentiating C2C12 myoblasts, terminally differentiated C2C12 myotubes, primary skeletal muscle cells or mature muscle. Both G-CSF and G-CSFR are dysregulated in many disease states. For example, ovarian cancers express G-CSFR (Ninci et al., 2000; Savarese et al., 2001), which contributes to cell survival and migration (Kumar et al., 2014), and bladder cancers express G-CSFR during the early stages of their development (Tachibana et al., 1997; Chakraborty et al., 2004, 2006), despite these tissues not normally expressing G-CSFR. In patients with ALS, G-CSF levels are elevated in the cerebrospinal fluid, while G-CSFR levels are decreased (Tanaka et al., 2006). In contrast, mouse models of ALS show increased levels of both and G-CSF/G-CSFR in neural tissue (Pitzer et al., 2008).

Treatment with G-CSF attenuates the severity of ischemic heart disease, acute myocardial infarction and chronic heart failure by stimulating the proliferation of cardiomyocytes (Li et al., 2006, 2007; Takano et al., 2006; Ueda et al., 2006). In skeletal muscle, G-CSF treatment improves force recovery, activates satellite cells and increases muscle mass following crush injury (Stratos et al., 2007). G-CSF treatment increases muscle repair following a local cardiotoxin (CTX) injection in mice (Naito et al., 2009) and increases muscle function in mouse models of amyotrophic lateral sclerosis (ALS) (Pitzer et al., 2008). Eight weeks of G-CSF treatment (30 μg/kg/day) increased Akt phosphorylation in neural tissue of ALS mice (Pitzer et al., 2008). G-CSF also increased Akt phosphorylation in regenerating skeletal muscle after myotoxic injury (Naito et al., 2009). These observations support a role for G-CSF in improving muscle health following injury. Therefore, understanding the molecular mechanisms for G-CSF action in skeletal muscle remains essential for implementing G-CSF as therapeutic agent for skeletal muscle disease.

The aims of this study were to firstly, to characterize the mRNA and protein expression of G-CSF and G-CSFR during muscle cell differentiation and in healthy and diseased mature skeletal muscle. The second aim was to determine the role of G-CSF in C2C12 myoblast differentiation and its ability to phosphorylate several proteins involved in muscle cells differentiation including Akt, STAT3, Erk-1, and Erk-2.

## Methods

### Tissue extractions

The human muscle biopsy procedure was approved by the Deakin University Human Research Ethics Committee in accordance with the Declaration of Helsinki (2000). Skeletal muscle samples were obtained under local anaesthesia (1% Xylocaine) from the belly of the *vastus lateralis* muscle using a percutaneous needle biopsy technique (Bergstrom, 1975), modified to include suction (Evans et al., 1982). Following a small incision through the skin, muscle biopsies were taken using a Bergstrom needle. Muscle samples were snap frozen in liquid nitrogen for RNA and protein extraction, with fresh muscle used for primary myoblast cultures.

The diaphragm and *tibialis anterior* (TA) muscles from 8 to 9 week old C57BL/10 and *mdx* mice were excised as part of a previously published study (Gehrig et al., 2012). Whole blood was obtained by cardiac puncture and immediately centrifuged at 4000 rpm for 5 min, with the upper plasma phase transferred to a sterile Eppendorf tube and frozen at −80°C. Male mice were used for all experiments. All experiments were approved by the Animal Ethics Comitte (AEC), The University of Melbourne and conducted in accordance with the Australian code of practice for the care and use of animals for scientific purposes, as stipulated by the National Health and Medical Research Council (Australia).

### Cell culture

For myoblast proliferation experiments, C2C12 myoblasts (American Type Culture Collection, ATCC, Manassas, VA) were seeded at a density of 50 cells per mm^2^ and incubated at 37°C, 5% CO_2_ in growth media consisting of high glucose Dulbecco's Modified Eagle's Medium (DMEM) supplemented with 10% (v/v) foetal bovine serum (FBS) (Invitrogen, Carlsbad, CA). For myotube differentiation experiments, C2C12 were seeded at a density of 150 cells per mm^2^ and incubated as above. G-CSF was used at concentrations between 0.4 and 100 ng/ml as published previously (Ward et al., 1999).

BAF/3[G] cells, a pro-B mouse cell line stably transfected with G-CSFR (Ward et al., 1999), were maintained at 37°C, 5% CO_2_ in DMEM containing 10% (v/v) FBS and 10% (v/v) conditioned WEHI-3B medium. HEK293T cells (ATCC) were maintained at 37°C, 5% CO_2_ in high glucose DMEM supplemented with 10% (v/v) FBS (Invitrogen). Human primary muscle cell cultures were established as previously described in our laboratory (Wallace et al., 2011).

#### Proliferation

Cells were allowed to attach overnight before being incubated with G-CSF at the desired concentration in DMEM supplemented with either 10% (v/v) FBS or 2% (w/v) BSA, which was replenished every 24 h. After 24, 48, 72, and 96 h C2C12 myoblasts were incubated with 0.1 μg/ml 4',6-Diamidino-2-phenylindole dihydrochloride (DAPI) (Sigma-Aldrich, Castle Hill, NSW, Australia) for 10 min at room temperature. Images were obtained using the Olympus Fluoview FV10i confocal laser scanning microscope with dedicated software at a 10 x magnification. A minimum of ten images per group were analyzed using ImageJ Software (National Institutes of Health, Bethesda, MA) to quantify the number of nuclei per image.

Myoblasts were assayed for DNA synthesis using a 5-bromo-2'-deoxyuridine (BrdU) Labeling and Detection Kit III (Roche, Castle Hill, NSW, Australia) according to the manufacturer's protocol. Briefly, following 24 and 48 h of BrdU labeling (10 μ M), myoblasts were fixed in 70% ethanol in 0.5 M HCl at −20°C for 30 min. Following a series of PBS washes containing 10% FBS, cells were incubated with the supplied nucleases at 37°C in the absence of CO_2_ for 30 min. Cells were again washed and incubated with anti-BrdU-POD Fab fragments for 30 min at 37°C. After a final series of washes, peroxidase substrate with substrate enhancer was added to the wells and the substrate left to develop in the dark for 5 min before the absorbance was determined at a wavelength of 405 nm.

#### Differentiation

When myoblasts were confluent, DMEM containing 2% HS ± G-CSF was added to induce differentiation. Images were obtained every 24 h for 4 days using an Olympus IX70 microscope (Olympus, Mt Waverly, VIC, Australia), and an attached DS-U3 microscope camera with NIS-Elements imaging software (Nikon Instruments Inc., Melville, NY) to assess differentiation. At the same time points RNA was extracted from the cells and RT-PCR was performed on genetic markers of differentiation. The media ± G-CSF was replenished every 24 h throughout the experiment.

### RNA extraction, reverse transcription and real time polymerase chain reaction (real-time PCR)

TRI-Reagent (Ambion Inc., Austin TX) was used to extract RNA from each cell culture sample or approximately 20–40 mg wet weight of muscle, with RNA concentrations determined using a NanoDrop® ND-1000 spectrophotometer (NanoDrop products, Wilmington, DE). The RNA was reverse transcribed (RT) to synthesize first strand cDNA using a high-capacity RNA-cDNA reverse transcription kit (Applied Biosystems, Foster City, CA). Each sample was incubated with one unit of ribonuclease H (RNAse H) (Invitrogen) at 37°C for 30 min following reverse transcription.

A mixture containing 0.5 × SYBR® Green PCR Master Mix (Applied Biosystems) and the forward and reverse primers for the gene of interest (Table 1) was added to 25 ng cDNA. Real-time polymerase chain reaction (Real-Time PCR was performed using a Stratagene Mx3000p QPCR System (Stratagene, La, CA) run by MxPro QPCR Software (Stratagene). All primers were used at 300 nM with an annealing temperature of 60°C.

**Table 1:** **Human and mouse PCR primer sequences**.

**Primer**	**Accession no**.	**Sense (5'–3')**	**Anti-sense (5'–3')**
*CSF3 (G-CSF)*	NM_000759	GAGTTGGGTCCCACCTTG	TGGAAAGCAGAGGCGAAG
*CSF3R (G-CSFR)*	NM_000760	CCTGCATCATCAAGCAGAAC	AGTTCAGGAAGCAGGAGAGA
*Csf3 (G-CSF)*	NM_009971	CGTTCCCCTGGTCAGTGTC	CCGCTGGCCTGGATCTTC
*Csf3r (G-CSFR)*	NM_007782	TCATGGCCACCAGTCGAGC	CACGCTGGAGTCCCAGAAG
*Myh7*	NM_080728	ACCCTCAGGTGGCTCCGAGA	TGCAGCCCCAAATGCAGCCA
*Myh2*	NM_001039545	GAGCAAAGATGCAGGGAAAG	TAAGGGTTGACGGTGACACA
*Myh4*	NM_010855	ACAGACTAAAGTGAAAGCC	CTCTCAACAGAAAGATGGAT
*Myh1*	NM_030679	GGACCCACGGTCGAAGTTGCA	GGAACTCATGGCTGCGGGCT

In some experiments, the resulting PCR products were separated on a 1.8% agarose gel containing 0.5 × SYBR® Safe DNA Gel Stain (Invitrogen). Images were captured using the Kodak Gel Logic 112 image station (Kodak Scientific Imaging Systems, Rochester, NY) under 302 nm UV light. The cDNA fragments were purified using the QIAquick Gel Extraction Kit (QIAGEN, Clifton Hill, VIC, Australia) according to the manufacturer's protocol. The purified cDNA from BAF/3[G] and C2C12 myoblasts and C2C12 myotubes was sequenced by the Australian Genome Research Facility (AGRF, Parkville, VIC, Australia).

### Protein extraction and western blot analysis

Myoblasts and myotubes were serum starved in DMEM (Invitrogen) for 4 h followed by stimulation with serum free media ± G-CSF. Total protein was extracted after 5, 10, 15, 30, and 90 min and phosphorylation was compared to basal levels following 4 h of serum starvation.

Total protein was extracted from muscle tissue and cell culture using 1x radioimmunoprecipitation (RIPA) buffer (Millipore, North Ryde, NSW) with 1 μL/mL protease inhibitor cocktail (Sigma-Aldrich, Castle Hill, NSW) and 10 μL/mL Halt Phosphatase Inhibitor Single-Use Cocktail (Thermo Scientific, Rockford, IL). Samples were centrifuged at 13,000 rpm for 15 min with the protein concentration of the supernatant determined from the development of a standard curve using the bicinchoninic acid (BCA) assay Protein Assay Kit (Pierce Biotechnology, Rockford, IL) according to the manufacturer's protocol.

Protein deglycosylation was performed using the PNGase F (New England Biolabs, Ipswich, MA) according to the manufacturer's protocol. In brief, 20 μg total protein was denatured in 1 × glycoprotein denaturing buffer at 100°C for 10 min. Following denaturing 1 × G7 reaction buffer, 2 μl 10% (v/v) NP-40 and 2 μl PNGase F was added to the protein sample. The sample was incubated using a water bath at 37°C for 1 h. The samples were frozen at −80°C for Western blot analysis.

Electrophoresis was performed using a 4–12% NuPAGE® Novex Bis-Tris Gel in NuPAGE® SDS MOPS Running Buffer (Invitrogen). Proteins were transferred to a PVDF membrane (Millipore) in a Bjerrum buffer containing 50 mM Tris, 17 mM glycine and 10% (v/v) methanol. The membranes were blocked with 5% BSA in PBS, after which they were incubated overnight at 4°C with anti-G-CSFR antibody (1:1000) (Santa Cruz Biotechnology, USA), anti-phospho-Akt (Ser473), anti-phospho-STAT3 (Tyr705), anti-phospho-Erk1/2 (Thr202/Tyr204), anti-Akt, anti-STAT3 and and anti-Erk1/2 (Cell Signaling Technology, Denvers, MA) diluted in PBS containing 5% BSA. Following washing, the membranes were incubated with goat anti-rabbit IgG antibody (1:5000) labeled with an infrared-fluorescent 800 nm dye (Alexa Fluor® 800, Invitrogen) in PBS containing 50% Odyssey ® blocking buffer (LI-COR Biosciences, Lincoln, USA) and 0.01% (w/v) SDS. After washing, the proteins were exposed on an Odyssey ® Infrared Imaging System (LI-COR). Anti-GAPDH antibody (1:5000) (G8795, Sigma-Aldrich, Australia) and rabbit anti-mouse IgG antibody labeled with an infrared-fluorescent 680 nm dye (Alexa Fluor® 680, Invitrogen) was used as a loading control.

### Cytokine analysis

A Milliplex assay (Millipore) was used to analyse G-CSF protein expression in the skeletal muscle and plasma samples following the manufacturer's protocol (Millipore). Briefly, the samples and standards were added to a 96 well plate containing premixed beads coated with G-CSF antibody. Following a 30 min incubation plates were washed three times then incubated with pre-mixed detection antibodies. Wells were then washed and incubated with streptavidin-phycoerythrin. After a final series of washes samples were resuspended in 125 μ l of assay buffer. The plate was read on the Bio-Plex Suspension Array System (V.5.0, Bio-Rad). For skeletal muscle, samples were extracted according to the protein extraction method described above and diluted to 1000 μg/ml prior to the assay. Plasma samples were run undiluted. Intra-assay coefficient of variation (CV%) was 4.2% for the plasma and 7.1% for the diaphragm homogenate.

### Statistical analysis

Statistical analysis was performed using GraphPad Prism 4.1 (GraphPad Software, San Diego, CA). Comparisons between wild type control and *mdx* mice were made using an unpaired Student t-test. Significance was set at *p* < 0.05 for all statistical tests with data represented as mean ± SEM.

For measurements of proliferation a One-Way ANOVA was used to determine the interaction between treatment concentration (0, 0.4, 4, 40, and 100 ng/ml G-CSF) at each time point. For BrdU analysis a Two-Way ANOVA was used to determine the interaction between G-CSF concentration (0, 0.4, 4, 40, and 100 ng/ml G-CSF) and treatment group (under normal growth conditions vs. serum depletion). Newman-Keuls Multiple Comparison Test was used to determine differences between groups with significance set at *p* < 0.05.

For measurements of differentiation a One-Way ANOVA was used to determine the interactions of time (day 1, 2, 3, and 4). Furthermore, a One-Way ANOVA was used to determine the interaction between treatment concentrations (0, 0.4, 4, 40, and 100 ng/ml G-CSF) at each day. Newman-Keuls Multiple Comparison Test was used to determine differences between groups with significance set at *p* < 0.05.

For densitometry analysis a One-Way ANOVA was used to determine the interaction of time (0, 5, 10, 15, 30, 60, and 90 min) at the given concentration of G-CSF treatment. Furthermore, a Two-Way ANOVA was used to determine an interaction between time (0, 5, and 15 min) and treatment (Control and G-CSF). Newman-Keuls Multiple Comparison Test was used to determine differences between groups with significance set at *p* < 0.05.

## Results

### Identification of G-CSFR in skeletal muscle

**Figure 1:**
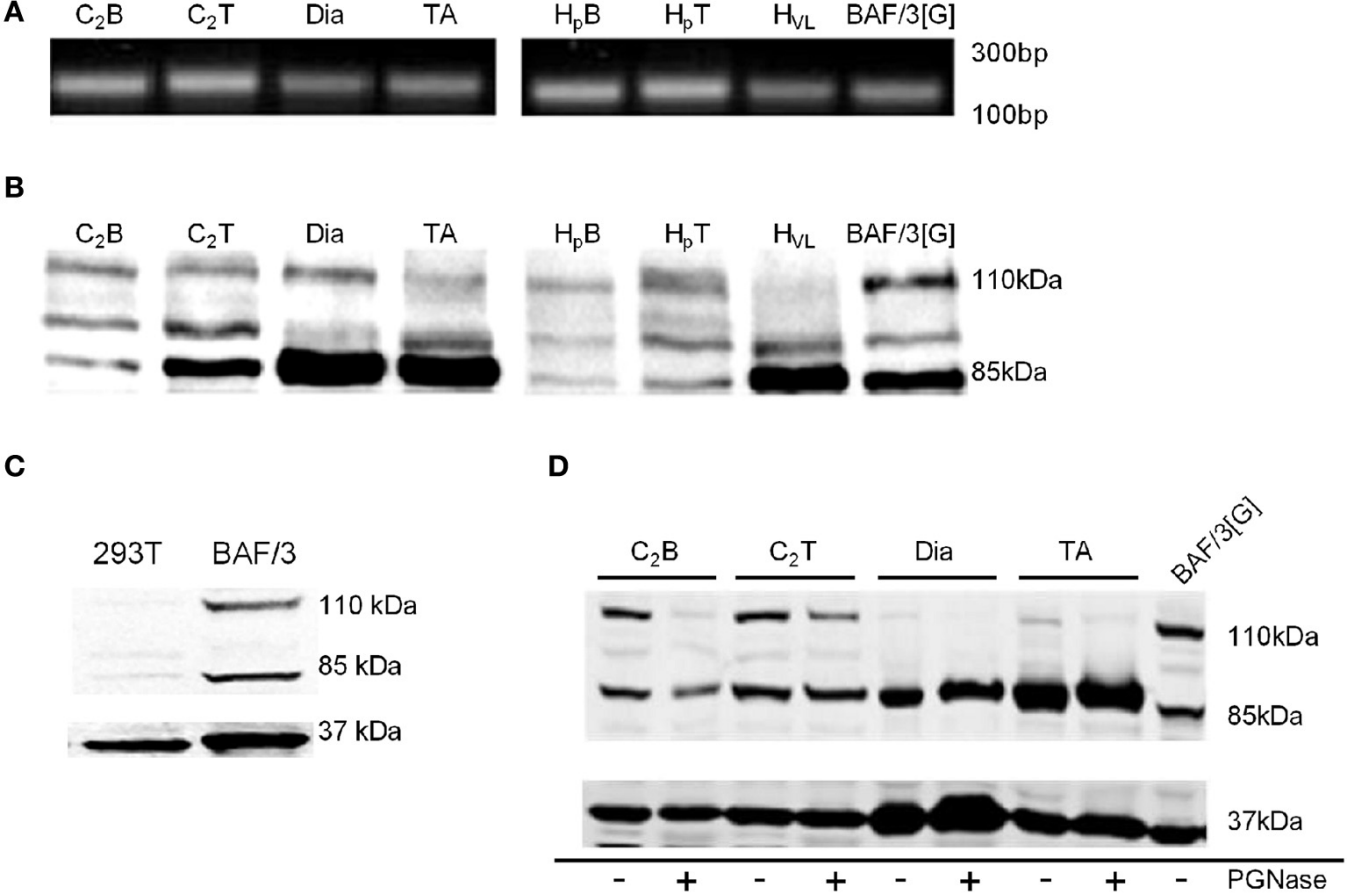
**Identification of G-CSFR in rodent and human skeletal muscle. (A)** cDNA fragment amplified during Real Time-PCR using the primers described in Table 1, separated on a 1.8% Sybr safe (Invitrogen) agarose gel and exposed to UV light. **(B)** Western blot image identifying G-CSFR in rodent and human skeletal muscle *in vitro* and *ex vivo*. **(C)** Western blot for G-CSFR in positive (BAF/3[G]) and negative (293T) control cells. **(D)** G-CSFR after deglycosylation with PGNase. C_2_B (C2C12 myoblasts), C_2_T (C2C12 myotubes), Dia (diaphragm muscle from C57BL/10 mice), TA (tibialis anterior muscle from C57BL/10 mice), H_p_B (human primary myoblasts), H_*p*_T (human primary myotubes) H_VL_ (human vastus lateralis muscle), BAF/3[G] (murine pro B cell line overexpressing G-CSFR) and 293T (human embryonic kidney 293T cell line). Experiments were performed in triplicate.

To investigate the expression of the genes encoding G-CSF and G-CSFR, intron-spanning primers described in Table 1 were used for Real-Time PCR. Fragments of the expected ~200 base pairs were observed in both mouse and human myoblasts, myotubes and tissue homogenates (Figure 1A). Sequencing of these products confirmed that they represented *bone fide* sequences, confirming expression of the G-CSFR in myoblasts and myotubes (Table S1).

Using a polyclonal antibody raised against amino acids 25–200 of human G-CSFR (Santa Cruz, Delaware, CA), Western blot analysis confirmed expression of G-CSFR protein in mouse C2C12 and human primary myoblasts and myotubes, as well as in mouse and human skeletal muscle (Figure 1B). Multiple bands were observed, consistent with multiple glycosylated forms of the receptor. Similar forms were observed in BAF/3 cells stably expressing the G-CSFR protein used as a positive control (Figures 1B,C), but not in HEK293T cells used as a negative control since they are known not to express G-CSFR (Debruin et al., 2010) (Figure 1C). Treatment of protein lysates with PGNase reduced the band intensity at 110 kDa in C2C12myoblasts, C2C12 myotubes and in mouse and human skeletal muscle homogenates, confirming the 110 kDa band as a glycosylated form of the G-CSFR protein (Figure 1D).

**Figure 2:**
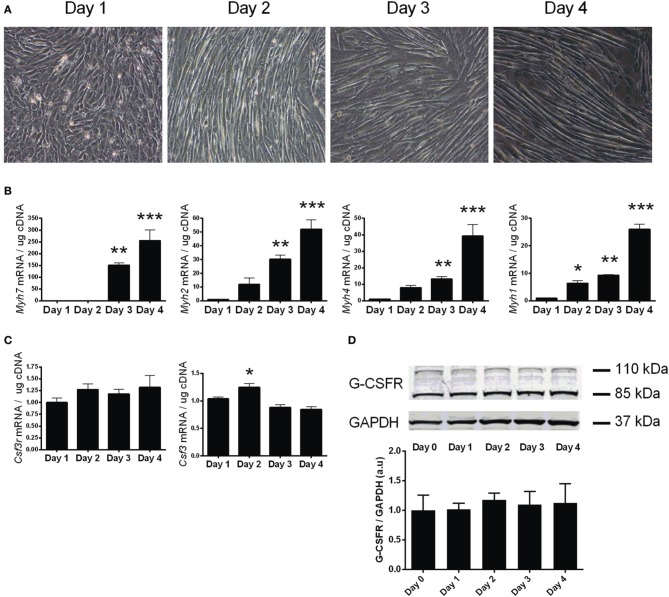
**Characterization of G-CSF and G-CSFR during C2C12 myogenesis. (A)** Representative images of C2C12 during differentiation. **(B)** Expression of myosin heavy chain genes (Myh7, 2, 4, 1) during following differentiation of C2C12 cultures. **(C)** G-CSF (*Csf3)* and G-CSFR (*Csf3r)* mRNA expression and **(D)** G-CSFR protein expression during differentiation of C2C12 cultures. Day 0 represents near confluent myoblasts (>90% confluent). Day 1 represents 24 h post serum withdrawal. mRNA is expressed as a fold change after Day 0 (*n* = 3). ^*^p < 0.05, ^**^p < 0.01 and ^***^p < 0.001.

To investigate the expression of the G-CSFR throughout myotube development, differentiation of confluent C2C12 myoblasts was induced by serum depletion. C2C12 cells formed visible myotubes after 4 days (Figure 2A), with differentiation confirmed by measuring various myosin heavy chain (*Myh7, 4, 2, 1*) mRNA levels, known markers of muscle cell differentiation (Brown et al., 2012). All *Myh* mRNAs were elevated by day 3 (*p* < 0.01) and day 4 (*p* < 0.001) when compared to day 0 (Figure 2B). G-CSF mRNA transiently increased by 20% (*p* < 0.05) at day 2 (Figure 2C), but no change in G-CSFR mRNA or protein levels was observed during differentiation (Figures 2C, D).

### The regulation of G-CSF and G-CSFR in muscles from *mdx mice*

**Figure 3:**
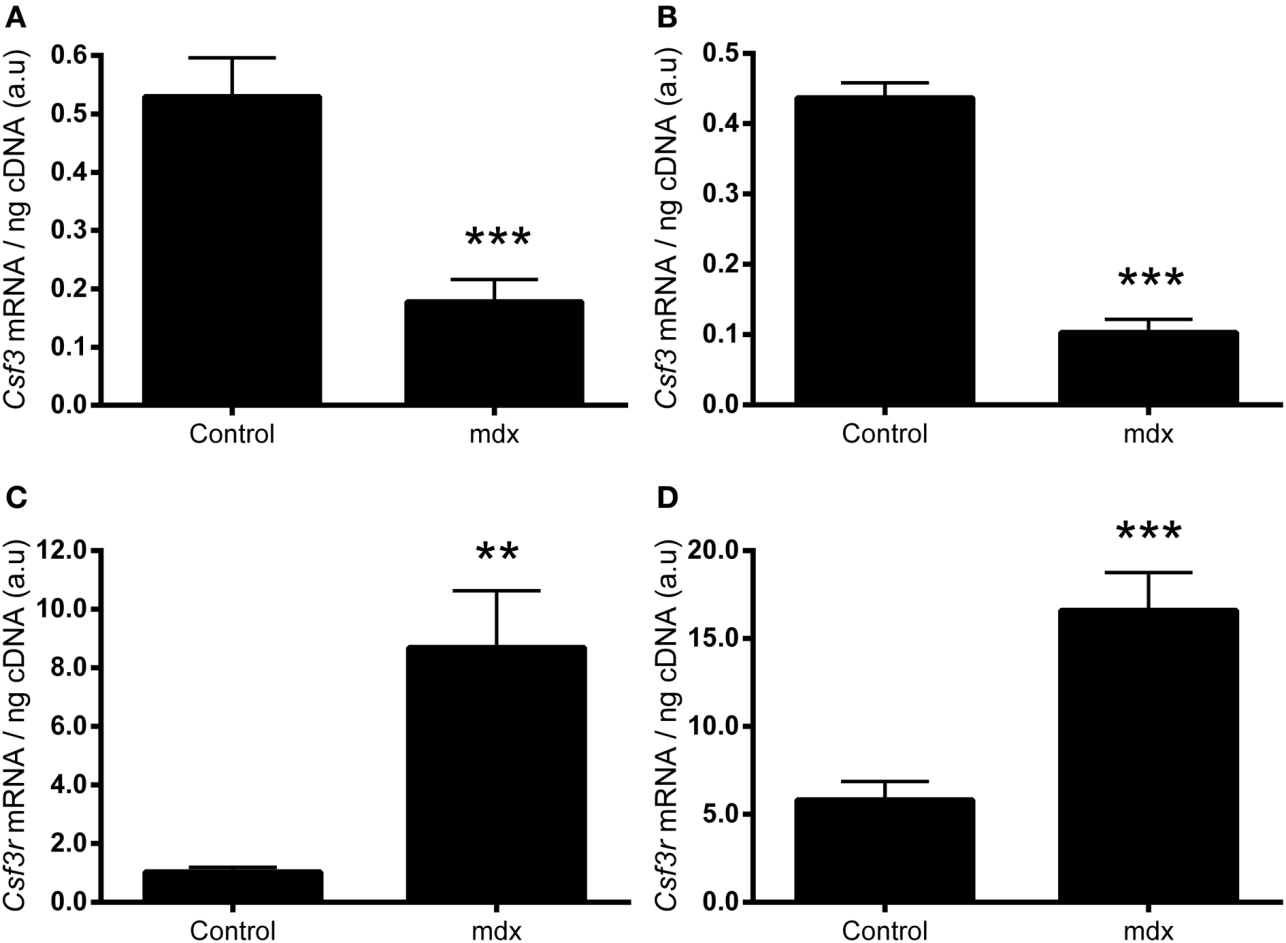
**mRNA expressions of G-CSF and G-CSFR mRNA in muscles from *mdx* mice. (A)** G-CSF (*Csf3*) mRNA in the *tibialis anterior* (TA) muscle. **(B)** G-CSF (*Csf3)* mRNA in the diaphragm. **(C)** G-CSFR (*Csf3r)* mRNA in the TA and **(D)** G-CSFR (*Csf3r)* mRNA in the diaphragm. mRNA was normalized to cDNA content. ^**^p < 0.01 and ^***^p < 0.001 (*n* = 10 per group).

**Figure 4:**
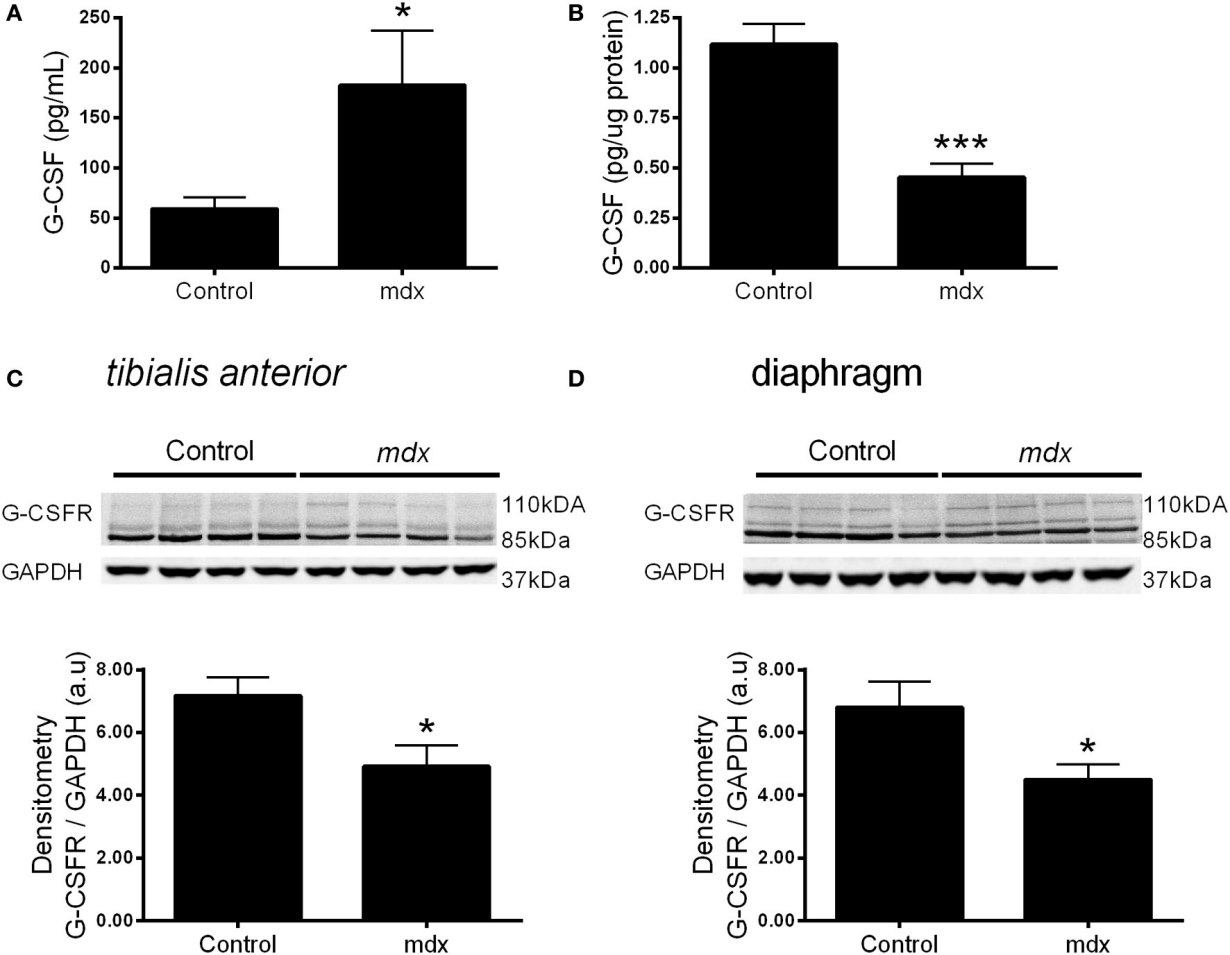
**G-CSF and G-CSFR protein expression in muscles from *mdx* mice. (A)** Circulating plasma G-CSF levels. **(B)** G-CSF levels in the diaphragm homogenate. **(C)** Representative western blot images for the G-CSFR in the tibialis anterior muscle and band densitometry analysis and **(D)** Representative western blot images for the G-CSFR in the diaphragm muscles from *mdx* mice and littermate controls and band densitometry analysis. ^*^p < 0.05 and ^***^p < 0.001. Cytokine analysis (*n* = 10), Western Blotting (*n* = 8) *NB*: G-CSF protein was undetectable in the TA muscle homogenate.

We next sought to examine whether the expression of G-CSF or its receptor were altered in dystrophic muscle, for which we used the *mdx* mouse. G-CSF mRNA was reduced by approximately 70% in the TA (*p* < 0.001) and approximately 80% in the diaphragm muscles (*p* < 0.001) of 8–9 week old *mdx* mice compared to littermate controls (Figures 3A,B). In contrast G-CSFR mRNA levels were 8-fold (*p* < 0.01) and >15-fold (*p* < 0.001) higher in the TA and diaphragm muscles, respectively, of *mdx* mice compared with control mice (Figures 3C,D). Circulating G-CSF levels were also significantly elevated in *mdx* mice compared with littermate controls (*p* < 0.05) (Figure 4A). However, the G-CSF protein was also reduced in the diaphragm muscle (*p* < 0.001) (Figure 4B), consistent with the G-CSF mRNA analysis. Levels of the G-CSF ligand were below the detectable limits of the assay in the TA muscle (data not shown). In contrast to levels of the G-CSFR mRNA, the G-CSFR protein was significantly reduced in both the TA (*p* < 0.05) and diaphragm muscle (*p* < 0.05) of *mdx* mice (Figures 4C,D).

### The role of G-CSF in proliferation, differentiation, and protein signaling in C2C12 myoblasts

**Figure 5:**
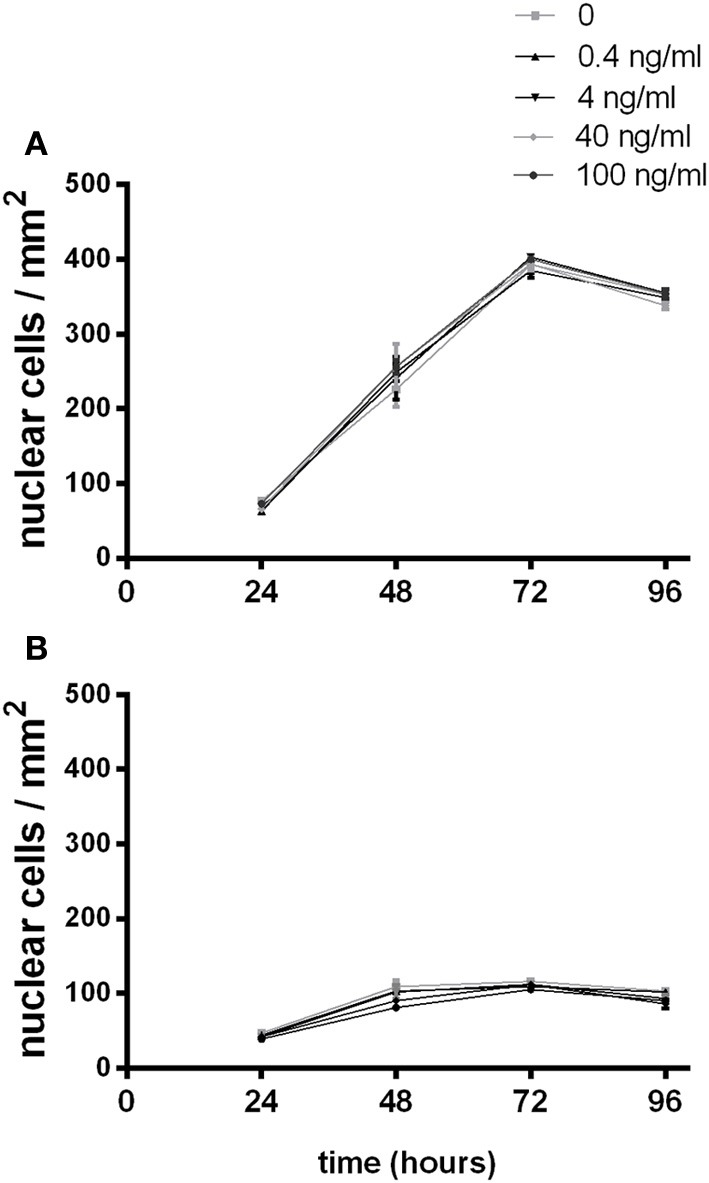
**The effects of G-CSF treatment on C2C12 myoblast proliferation**. Proliferating C2C12myoblasts incubated in the presence of the indicated concentration of G-CSF under **(A)** normal growth media containing 10% fetal bovine serum or **(B)** serum depleted growth media containing 2% BSA. Data represents the mean ± SEM taken from a minimum of 10 images per sample (*n* = 3).

To investigate the effects of G-CSF on myoblast proliferation, myoblasts were enumerated by DAPI staining at 24 h intervals over 96 h (Supplementary Figures 1, 2). C2C12 myoblasts proliferated as expected, approximately doubling at 48 and 72 h, reaching confluency and showing signs of spontaneous fusion at 96 h under normal growth conditions (Figure 5A). Slower proliferation rates were observed in serum depleted conditions over the first 48 h followed by the cessation of proliferation thereafter (Figure 5B). No changes in cell number was observed with the addition of G-CSF up to 100 ng/ml under normal or serum depleted conditions (Figure 5).

To confirm that G-CSF was not affecting proliferation, DNA synthesis was assayed by BrdU incorporation in C2C12 myoblasts for 24 and 48 h. In normal growth conditions C2C12 myoblasts incorporated more BrdU into their cellular DNA compared to serum depleted cells. G-CSF treatment did not have any effect on BrdU incorporation at 24 and 48 h (Supplementary Figure 3)

**Figure 6:**
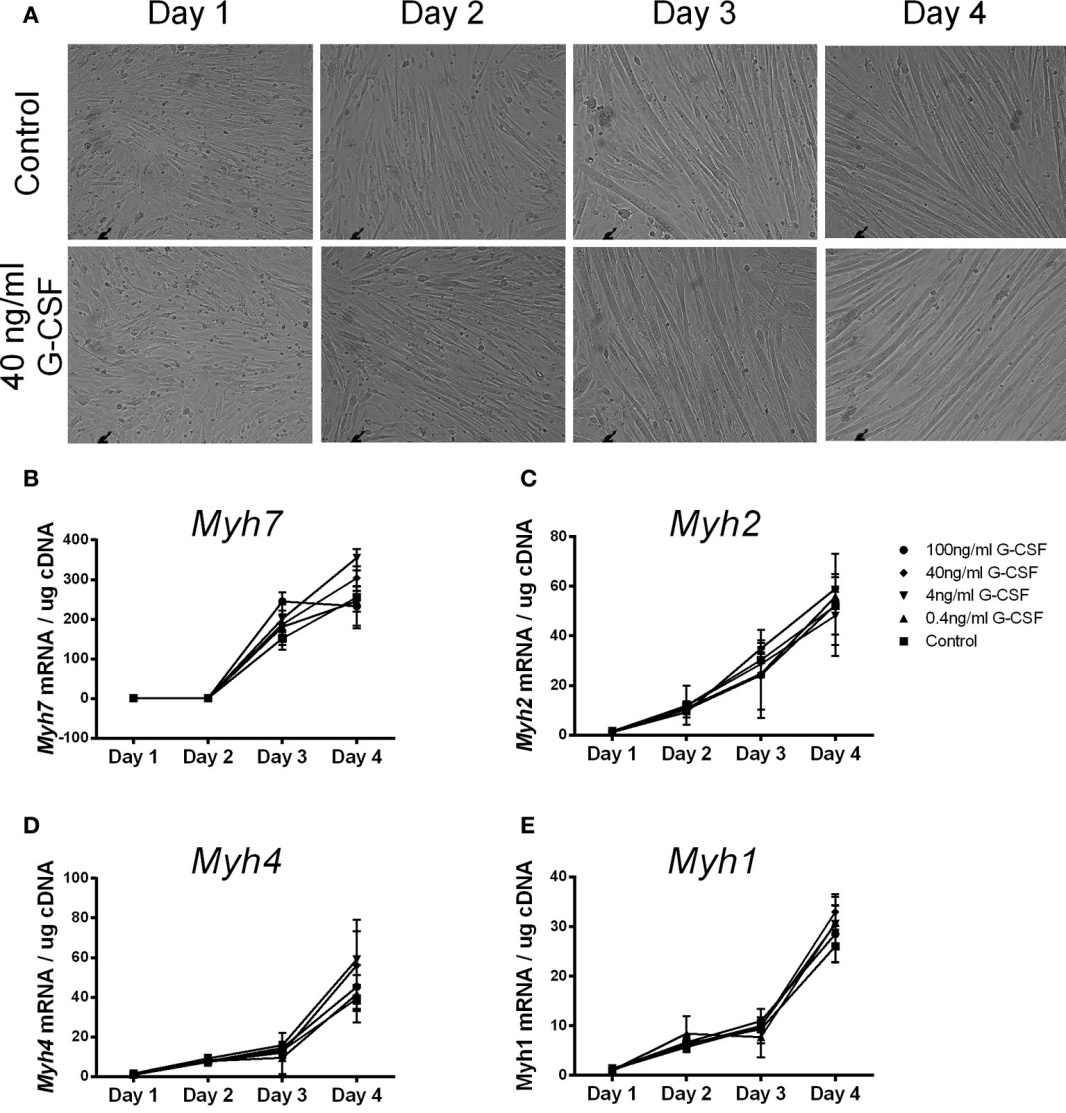
**The effects of G-CSF treatment on expression of *Myh* genes during C2C12 cell differentiation. (A)** Light microscope images of C2C12 cells at 24 h intervals during differentiation under normal conditions and in the presence of 40 ng/ml G-CSF. **(B)** *Myh7* **(C)** *Myh2* **(D)** *Myh4*, and **(E)** *Myh1* mRNA expression during 4 days of differentiation with 0, 0.4, 4, 40, and 100 ng/ml G-CSF. Data is represented as mean ± SEM of the fold change compared to the Day 1 control (*n* = 3).

To examine the effects of G-CSF on myoblast differentiation G-CSF was added to C2C12 myoblasts during myotube formation. C2C12 cells formed visible myotubes after 4 days in the absence of G-CSF, as expected, with no visible differences observed in G-CSF treated cells (Figure 6A). G-CSF also had no effect on the level of expression of *Myh7*, *Myh4, Myh2*, and *Myh1* which all increased during differentiation (Figures 6B–E), nor on the myogenic regulatory factors MyoD and myogenin during C2C12 differentiation (data not shown).

**Figure 7:**
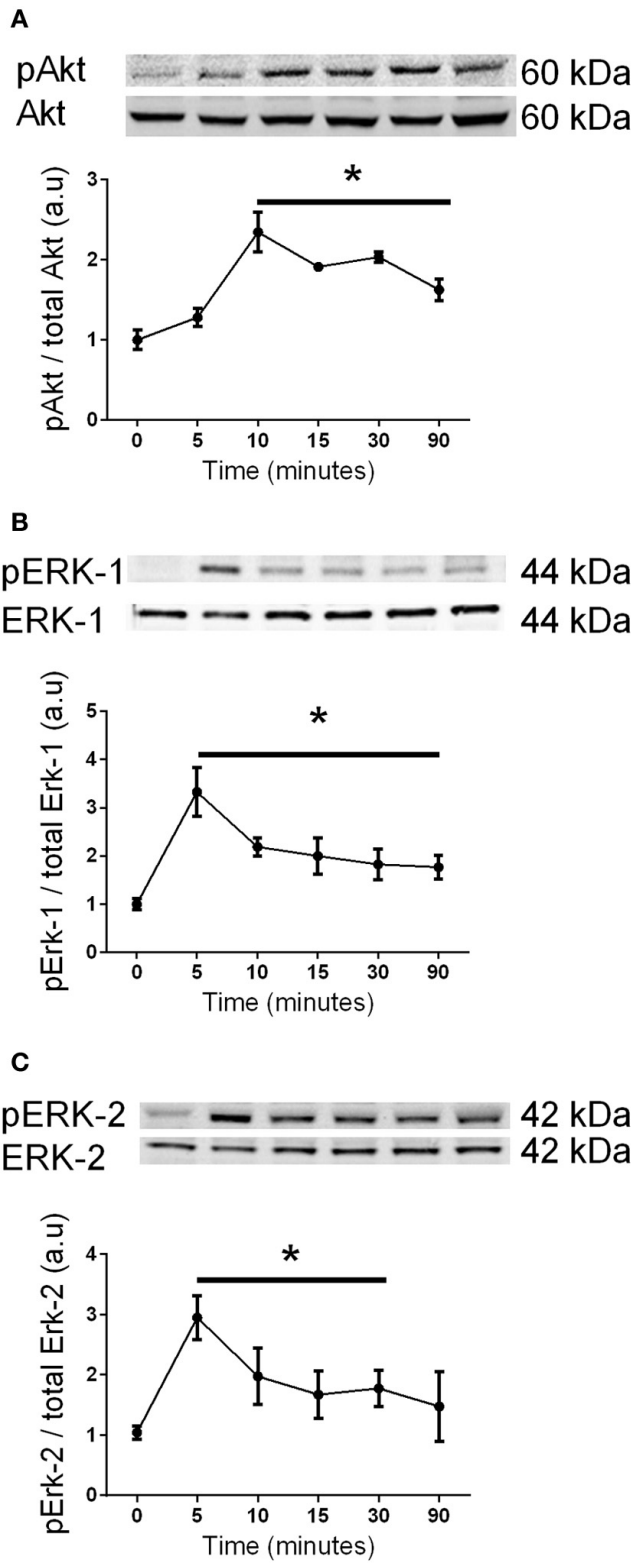
**The effects of changing media on Akt and Erk1/2 phosphorylation in C2C12 myoblasts**. Western blot analysis using phospho-specific and control antibodies for **(A)** Akt, **(B)** Erk-1, and **(C)** Erk-2 after replenishing the media. Each panel shows a representative western blot above a graph of the fold-change, presented as mean ± SEM compared to time zero (*n* = 3, ^*^p < 0.05).

**Figure 8:**
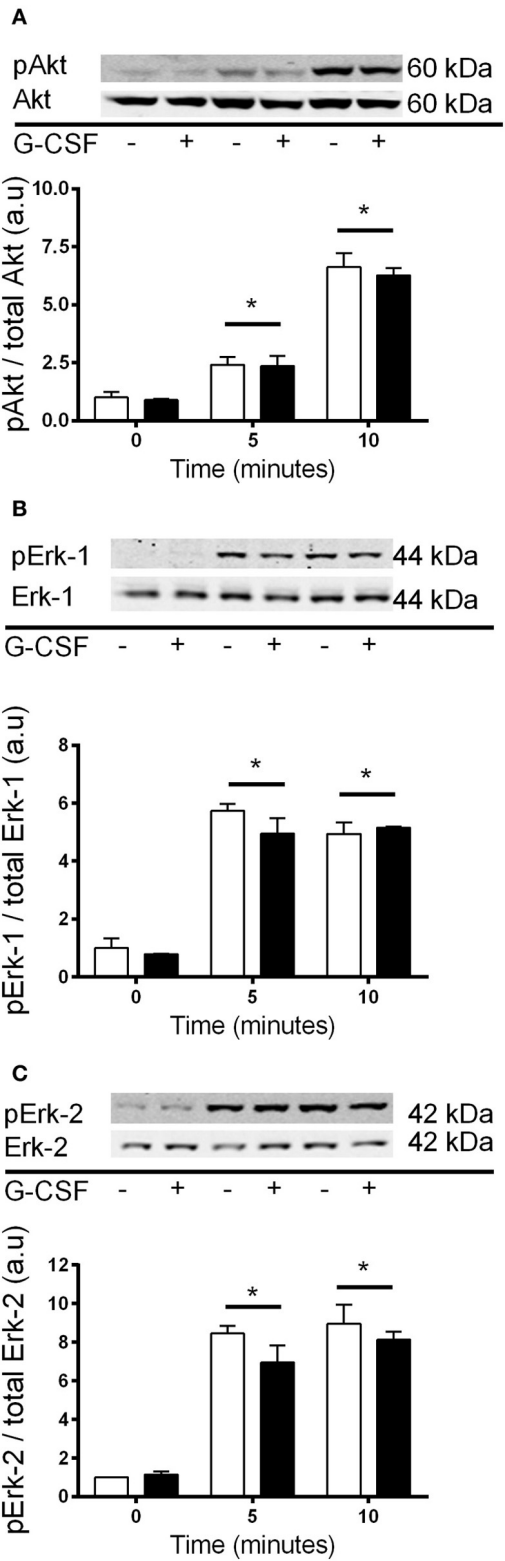
**Akt and Erk1/2 phosphorylation with 40 ng/ml G-CSF vs. vehicle treatment in myoblasts**. Fold changes in phosphorylation for **(A)** Akt **(B)** Erk-1, and **(C)** Erk-2 after treatment with fresh media containing 40 ng/ml G-CSF (black bars) or vehicle control (white bars). Data is shown as mean ± SEM compared to time zero (*n* = 3, ^*^p < 0.05).

**Figure 9:**
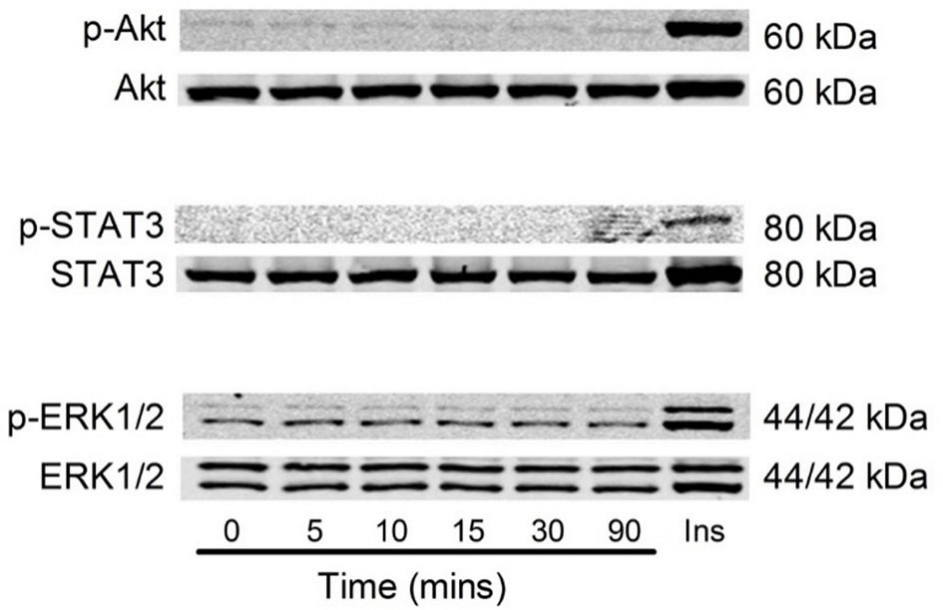
**Akt, STAT3 and Erk1/2 phosphorylation with 40 ng/ml G-CSF in C2C12 myoblasts**. Representative images of Akt, STAT3, and Erk1/2 phosphorylation following stimulation with G-CSF (40 ng/ml) from 5 to 90 min. 20 μ l G-CSF was added to the media at time 0 min. Time zero represents 4 h after serum free media was added to the cells. Ins = C2C12 myoblast treated with 100 μ M insulin for 15 min using the spike in technique (*n* = 3).

It has previously been shown that Akt and Erk1/2 phosphorylation were sensitive to the stress associated with changing media in cardiomyocytes (Sinclair et al., 2010). We also examined this in C2C12 myoblasts, and observed that media changes also led to phosphorylation of both Akt and Erk1/2 in these cells (Figure 7). Therefore, to overcome the potential confounding effect of replenishing the media we compared cells treated with fresh media containing G-CSF (40 ng/ml) to those treated with fresh media containing vehicle only. G-CSF did not increase Akt or Erk1/2 phosphorylation above that of the vehicle treated cells (Figure 8). To confirm G-CSF was not affecting intracellular signaling protein in C2C12 myoblasts we used a “spike in” method in which 20 μ l G-CSF was added directly to the well containing serum free media to a final concentration of 40 ng/ml. When G-CSF was “spiked in” no changes in Akt, STAT3, or Erk1/2 phosphorylation were observed, in contrast to an insulin control (Figure 9).

## Discussion

G-CSF treatment improves recovery of mouse skeletal muscle after crush injury or myotoxic damage and improves muscle function in mouse models of ALS (Stratos et al., 2007; Pitzer et al., 2008; Hara et al., 2011). However, the molecular mechanisms responsible for this improvement in muscle function are unknown. G-CSFR expression has been observed on various cell types outside of the haematopoietic system leading to speculation that G-CSFR may be expressed in skeletal muscle. Consequently, G-CSF may directly bind to G-CSFR activating intramuscular signaling pathways involved in tissue repair. Therefore, the present study aimed to establish and characterize G-CSF and G-CSFR expression in proliferating, differentiating or fully differentiated C2C12 muscle cells, human primary muscle cells and in mature mouse and human muscle. Since G-CSF and G-CSFR levels are altered in various diseases (Ninci et al., 2000; Chakraborty and Guha, 2007; Pitzer et al., 2008, 2010; Hsu et al., 2013), the study also sought to characterize whether G-CSF and G-CSFR expressed was perturbed in dystrophic skeletal muscle. Lastly, we sought to elucidate the molecular mechanisms by which G-CSF exerts its effects in C2C12 myotubes. G-CSFR expression was confirmed in C2C12 myoblasts with several novel observations. Firstly, G-CSFR mRNA and protein was identified in mouse C2C12 and human primary myoblasts and myotubes and in mature skeletal muscle. Secondly, G-CSF mRNA was down-regulated in the diaphragm and TA muscles, while protein expression was down-regulated in the diaphragm and indeed undetectable in the TA muscles of *mdx* mice compared with wild type mice. In contrast, the G-CSFR mRNA levels were increased, while G-CSFR protein levels were decreased in both the diaphragm and the TA muscles of *mdx* mice. Lastly, G-CSF treatment of C2C12 muscle cells did not increase proliferation, differentiation or intracellular activation of Akt, STAT3, and Erk1/2.

The identification of G-CSFR outside of tissues of haematopoietic origin has significant clinical relevance. Identifying G-CSFR in cardiac myocytes (Harada et al., 2005) has seen the administration of recombinant G-CSF progress from rodent models (Li et al., 2006; Takano et al., 2006, 2007; Ueda et al., 2006) to human clinical trials as a therapy to combat cardiac remodeling post infarction (Takano et al., 2007). The present study identified G-CSFR expression at both the mRNA and protein levels in proliferating C2C12 myoblasts, as well as in terminally differentiated C2C12 myotubes, human primary skeletal myoblast and myotube cultures and mature skeletal muscles from mice and humans. Our results support a recent report that G-CSFR protein is expressed in proliferating C2C12 myoblasts (Hara et al., 2011), although there were also several important differences with that study. We show that G-CSFR is expressed in both myoblasts and myotubes, as well as muscle tissue, with multiple protein bands detected between 85 kDa and 110 kDa by Western blot. In contrast, Hara et al. detected a single band for the G-CSFR at 110 kDa in C2C12 myoblasts but not in differentiated myotubes (Hara et al., 2011). Moreover, positive and negative controls known to express/not express the G-CSFR were not used to show antibody specificity (Hara et al., 2011). This is important, since many G-CSFR antibodies have been proven to lack specificity in Western blotting and immunofluorescence (Debruin et al., 2010). In support of our data, similar multiple G-CSFR bands have been observed in mouse bone marrow which highly expresses G-CSFR (Hermans et al., 1998), and in our positive control, the murine pro-B cell line overexpressing G-CSFR. In addition, expression was confirmed by RT-PCR followed by sequencing of the product. This demonstrates definitively that G-CSFR is expressed in myoblasts, as well as differentiating and terminal myotubes, suggesting that G-CSF may have potential to aid in muscle regeneration as well as protecting against muscle loss by acting on mature muscle fibers.

Both G-CSF and G-CSFR are modulated in neural tissue of several disease models characterized with muscle atrophy and dysfunction, such as ALS patients and rodent models of ALS and spinal cord injury (Pitzer et al., 2008, 2010; Kawabe et al., 2011). Similar to these models, we observed perturbations in the expression of G-CSF and G-CSFR in the muscles of *mdx* mice, a widely studied model for human DMD (Schertzer et al., 2008; Stupka et al., 2008). In *mdx* mice, G-CSF mRNA was reduced in the TA muscles and diaphragm, while G-CSF protein was reduced in the diaphragm. G-CSFR expression was increased, as shown by mRNA. However, plasma G-CSF was raised, probably as a result of inflammation induced by the muscle damage (Hirose et al., 2004; Paulsen et al., 2005). This can ligate with its receptor, thereby degrading it in the lysosome and proteasome (Irandoust et al., 2007; Kindwall-Keller et al., 2008), leading to a decrease in G-CSFR protein. One corollary of this scenario would be that perhaps G-CSFR is already providing protective signals in the *mdx* mouse, with symptoms potentially worsened if G-CSFR was ablated. However, the importance of these observations remains unknown and warrants further investigation. G-CSF treatment in ALS mice increases muscle mass, muscle function and survival (Pitzer et al., 2008). ALS mice have increased G-CSF and G-CSFR levels in neural tissue suggesting their elevation may be an attempted “survival” mechanism for damaged neurons and for subsequent preservation of neural innervated muscles.

The present study demonstrated that G-CSF does not stimulate C2C12 myoblast proliferation in the presence or absence of serum. Importantly, our C2C12 myoblasts doubled approximately every 24 h in serum, and exhibited a block in proliferation without serum, consistent with other studies (Yaffe and Saxel, 1977). In contrast, in the study by Hara et al showing G-CSF treatment increased C2C12 myoblast proliferation they observed a reduction in myoblast cell number in the first 48 h in the control group (Hara et al., 2011), which may indicate cell cycle arrest and induction of differentiation (Walsh and Perlman, 1997). They also did not clearly describe if proliferation was measured in the presence or absence of serum, nor was the level of cell confluence at the time of treatment with G-CSF stated (Hara et al., 2011), making their results difficult to interpret. Myosin heavy chain proteins regulate skeletal muscle contraction and their mRNA levels are significantly increased during myogenesis (Brown et al., 2012). In the current study mRNA expression levels for myosin heavy chains (*Myh*-7, -*4*, -*2* and -*1*) continued to increase throughout differentiation. G-CSF had no influence on the mRNA levels of these genes at any time point nor did G-CSF influence the myogenic regulatory factors MyoD and myogenin. This further supports our observation that G-CSF does not increase C2C12 myoblast proliferation or differentiation.

G-CSF is known to activate Akt, STAT3, and Erk1/2 in cells expressing the G-CSFR (Chakraborty and Tweardy, 1998; Dong and Larner, 2000). Serum starving (3–20 h) prior to experimental treatment is used routinely to reduce basal cellular activity (Rommel et al., 2001; Stitt et al., 2004). Following re-stimulation with fresh media with and without G-CSF there was a significant increases in protein phosphorylation. However, there was no difference between the vehicle control and the G-CSF treated groups, demonstrating the media changes alone were sufficient to produce these changes. To confirm G-CSF was not affecting protein phosphorylation, G-CSF was spiked into the media so that the media was not removed from the cells. This alternative approach also revealed that G-CSF did not increase Akt, STAT3, or Erk1/2 phosphorylation in C2C12myoblasts. Without the necessary controls, the increased protein phosphorylation observed following media change could be mistaken as an effect of G-CSF treatment, especially given the strong rationale and prior observations (Hara et al., 2011). However, the results of the present study show that a media change alone is sufficient to stimulate Akt, STAT3, and Erk1/2, indicating that G-CSF does not activate these signaling proteins in C2C12 muscle cells.

In conclusion, we show that the G-CSFR is expressed not just in myoblasts, but also in differentiated C2C12 and human primary myotubes and mature mouse and human muscles. These findings suggest that the G-CSF ligand may act directly on skeletal muscle via its receptor. We also show that G-CSF and the G-CSFR are reduced in the TA and diaphragm muscles from *mdx* mice. The relevance of altered G-CSF/G-CSFR in the muscles of *mdx* mice needs to be investigated to determine a causative, passive role in disease progression or whether G-CSF/G-CSFR is actively signaling to cause reduced G-CSFR levels. However, since G-CSF treatment increases muscle recovery in various rodent models of trauma and/or disease, a reduction in G-CSF within the muscle suggests G-CSF may be protective against damage, or that elevating intramuscular G-CSF promote muscle regeneration. Therefore, G-CSF may have therapeutic potential for managing the pathophysiology of muscular dystrophy.

The molecular mechanisms by which G-CSF exerts its effects remain elusive as the current study demonstrates that G-CSF does not increase myoblast proliferation with concentrations ranging from 0.4 to 100 ng/ml. The actions of G-CSF may therefore require the interaction with other cytokines and growth factors *in vivo*. For example G-CSF is known to cooperate with stem cell factor in haematopoiesis (Duarte and Franf, 2002) and IL-6 during bone marrow stem cell tumour progression (Yan et al., 2013). Alternatively, it may act directly on other cell populations, such as immune cells to stimulate the release of factors that are able to act directly on muscle cells. Furthermore, we showed that G-CSF does not increase Akt, STAT3, or Erk1/2 above that caused by replenishing media. This highlights the importance of using rigorous controls, meaning those studies lacking such controls should be interpreted with caution.

## Conflict of interest statement

The authors declare that the research was conducted in the absence of any commercial or financial relationships that could be construed as a potential conflict of interest.

## Supplementary material

Supplementary Figure 1 **BrDU incorporation into proliferating C2C12 myoblasts following G-CSF treatment**. Proliferating C2C12 myoblasts in Growth Media (GM) containing DMEM + 10% FBS (black bars) or DMEM + 2% BSA (white bars) following 0, 0.4, 4, 40, and 100 ng/ml G-CSF treatment for (1) 24 h and (2) 48 h. Data is Mean ± SEM, *n* = 8, ^*^*p* < 0.05 compared to GM.

Supplementary Figure 2 **DAPI staining of proliferating C2C12 myoblast following G-CSF stimulation grown in 10% FBS**. Representative images following DAPI staining of C2C12myoblastsfollowing 24, 48, 72, and 96 h in DMEM + 10% FBS with the indicated concentration of G-CSF. A minimum of 10 images were obtained per sample (*n* = 3).

Supplementary Figure 3 **DAPI staining of proliferating C2C12 myoblasts following G-CSF treatment with serum depletion**. Representative images following DAPI staining of C2C12myoblastsfollowing 24, 48, 72, and 96 h in DMEM + 2% BSA with the indicated concentration of G-CSF. A minimum of 10 images were obtained per sample (*n* = 3).

## References

[B1] Bergstrom J. (1975). Percutaneous needle biopsy of skeletal muscle in physiological and clinical research. Scand. J. Clin. Lab. Invest. 35, 609–616 10.3109/00365517509095787 1108172

[B2] Brown D. M. Parr T. Brameld J. M. (2012). Myosin heavy chain mRNA isoforms are expressed in two distinct cohorts during C2C12 myogenesis. J. Muscle Res. Cell Motil. 32, 383–390 10.1007/s10974-011-9267-4 22012579

[B3] Chakraborty A. Guha S. (2007). Granulocyte colony-stimulating factor/granulocyte colony-stimulating factor receptor biological axis promotes survival and growth of bladder cancer cells. Urology 69, 1210–1215 10.1016/j.urology.2007.02.035 17572226

[B4] Chakraborty A. Tweardy D. J. (1998). Stat3 and G-CSF-induced myeloid differentiation. Leuk. Lymphoma 30, 433–442 9711905 10.3109/10428199809057555

[B5] Chakraborty A. White S. M. Guha S. (2006). Granulocyte colony-stimulating receptor promotes beta1-integrin-mediated adhesion and invasion of bladder cancer cells. Urology 68, 208–213 10.1016/j.urology.2006.01.046 16844458

[B6] Chakraborty A. White S. M. Lerner S. P. (2004). Granulocyte colony-stimulating factor receptor signals for beta1-integrin expression and adhesion in bladder cancer. Urology 63, 177–183 10.1016/S0090-4295(03)00786-6 14751388

[B7] Dal-Re R. (2011). Worldwide clinical interventional studies on leading causes of death: a descriptive analysis. Ann. Epidemiol. 21, 727–731 10.1016/j.annepidem.2011.03.010 21550817

[B8] Debruin C. Lincoln P. Hartley C. Shehabeldin A. Van G. Szilvassy S. J. (2010). Most purported antibodies to the human granulocyte colony-stimulating factor receptor are not specific. Exp. Hematol. 38, 1022–1035 10.1016/j.exphem.2010.07.011 20696205

[B9] Dong F. Larner A. C. (2000). Activation of Akt kinase by granulocyte colony-stimulating factor (G-CSF): evidence for the role of a tyrosine kinase activity distinct from the Janus kinases. Blood 95, 1656–1662 10688821

[B10] Duarte R. F. Franf D. A. (2002). The synergy between stem cell factor (SCF) and granulocyte colony-stimulating factor (G-CSF): molecular basis and clinical relevance. Leuk. Lymphoma 43, 1179–1187 10.1080/10428190290026231 12152985

[B11] Evans W. J. Phinney S. D. Young V. R. (1982). Suction applied to a muscle biopsy maximizes sample size. Med. Sci. Sports Exerc. 14, 101–102 7070249

[B12] Gehrig S. M. van der Poel C. Sayer T. A. Schertzer J. D. Henstridge D. C. Church J. E. (2012). Hsp72 preserves muscle function and slows progression of severe muscular dystrophy. Nature 484, 394–398 10.1038/nature10980 22495301

[B13] Hara M. Yuasa S. Shimoji K. Onizuka T. Hayashiji N. Ohno Y. (2011). G-CSF influences mouse skeletal muscle development and regeneration by stimulating myoblast proliferation. J. Exp. Med. 208, 715–727 10.1084/jem.20101059 21422169 PMC3135344

[B14] Harada M. Qin Y. Takano H. Minamino T. Zou Y. Toko H. (2005). G-CSF prevents cardiac remodeling after myocardial infarction by activating the Jak-Stat pathway in cardiomyocytes. Nat. Med. 11, 305–311 10.1038/nm1199 15723072

[B15] Hermans M. H. Ward A. C. Antonissen C. Karis A. Lowenberg B. Touw I. P. (1998). Perturbed granulopoiesis in mice with a targeted mutation in the granulocyte colony-stimulating factor receptor gene associated with severe chronic neutropenia. Blood 92, 32–39 9639496

[B16] Hirose L. Nosaka K. Newton M. Laveder A. Kano M. Peake J. (2004). Changes in inflammatory mediators following eccentric exercise of the elbow flexors. Exerc. Immunol. Rev. 10, 75–90 15633588

[B17] Hsu D. M. Agarwal S. Benham A. Coarfa C. Trahan D. N. Chen Z. (2013). G-CSF receptor positive neuroblastoma subpopulations are enriched in chemotherapy-resistant or relapsed tumors and are highly tumorigenic. Cancer Res. 10.1158/0008-5472.CAN-12-4056 23687340 PMC4298227

[B18] Irandoust M. I. Aarts L. H. Roovers O. Gits J. Erkeland S. J. Touw I. P. (2007). Suppressor of cytokine signaling 3 controls lysosomal routing of G-CSF receptor. EMBO J. 26, 1782–1793 10.1038/sj.emboj.7601640 17363902 PMC1847666

[B19] Jagoe R. T. Goldberg A. L. (2001). What do we really know about the ubiquitin-proteasome pathway in muscle atrophy? Curr. Opin. Clin. Nutr. Metab. Care 4, 183–190 10.1097/00075197-200105000-00003 11517350

[B20] Kawabe J. Koda M. Hashimoto M. Fujiyoshi T. Furuya T. Endo T. (2011). Neuroprotective effects of granulocyte colony-stimulating factor and relationship to promotion of angiogenesis after spinal cord injury in rats: laboratory investigation. J. Neurosurg. Spine 15, 414–421 10.3171/2011.5.SPINE10421 21721873

[B21] Kindwall-Keller T. L. Druhan L. J. Ai J. Hunter M. G. Massullo P. Loveland M. (2008). Role of the proteasome in modulating native G-CSFR expression. Cytokine 43, 114–123 10.1016/j.cyto.2008.04.015 18554923 PMC2556513

[B22] Kirsch F. Kruger C. Schneider A. (2008). The receptor for granulocyte-colony stimulating factor (G-CSF) is expressed in radial glia during development of the nervous system. BMC Dev. Biol. 8:32 10.1186/1471-213X-8-32 18371196 PMC2329616

[B23] Kumar J. Fraser F. W. Riley C. Ahmed N. McCulloch D. R. Ward A. C. (2014). Granulocyte colony-stimulating factor receptor signalling via Janus kinase 2/signal transducer and activator of transcription 3 in ovarian cancer. Br. J. Cancer 110, 133–145 10.1038/bjc.2013.673 24220695 PMC3887286

[B24] Li L. Takemura G. Li Y. Miyata S. Esaki M. Okada H. (2007). Granulocyte colony-stimulating factor improves left ventricular function of doxorubicin-induced cardiomyopathy. Lab. Invest. 87, 440–455 10.1038/labinvest.3700530 17334414

[B25] Li Y. Takemura G. Okada H. Miyata S. Esaki M. Maruyama R. (2006). Treatment with granulocyte colony-stimulating factor ameliorates chronic heart failure. Lab. Invest. 86, 32–44 10.1038/labinvest.3700367 16304579

[B28] Murray C. J. Lopez A. D. (1997). Mortality by cause for eight regions of the world: global burden of disease study. Lancet 349, 1269–1276 10.1016/S0140-6736(96)07493-4 9142060

[B29] Nagata S. Fukunaga R. (1991). Granulocyte colony-stimulating factor and its receptor. Prog. Growth Factor Res. 3, 131–141 10.1016/S0955-2235(05)80004-3 1723014

[B30] Naito T. Goto K. Morioka S. Matsuba Y. Akema T. Sugiura T. (2009). Administration of granulocyte colony-stimulating factor facilitates the regenerative process of injured mice skeletal muscle via the activation of Akt/GSK3alphabeta signals. Eur. J. Appl. Physiol. 105, 643–651 10.1007/s00421-008-0946-9 19048276

[B31] Ninci E. B. Brandstetter T. Meinhold-Heerlein I. Bettendorf H. Sellin D. Bauknecht T. (2000). G-CSF receptor expression in ovarian cancer. Int. J. Gynecol. Cancer 10, 19–26 10.1046/j.1525-1438.2000.99076.x 11240647

[B32] Oishi A. Otani A. Sasahara M. Kojima H. Nakamura H. Yodoi Y. (2008). Granulocyte colony-stimulating factor protects retinal photoreceptor cells against light-induced damage. Invest. Ophthalmol. Vis. Sci. 49, 5629–5635 10.1167/iovs.08-1711 18676635

[B33] Paulsen G. Benestad H. B. Strã,M-Gundersen I. Mã,Rkrid L. Lappegã¥Rd K. T. Raastad T. (2005). Delayed leukocytosis and cytokine response to high-force eccentric exercise. Med. Sci. Sports Exerc. 37, 1877–1883 10.1249/01.mss.0000177064.65927.98 16286856

[B34] Piscaglia A. C. Shupe T. D. Oh S.-H. Gasbarrini A. Petersen B. E. (2007). Granulocyte-colony stimulating factor promotes liver repair and induces oval cell migration and proliferation in rats. Gastroenterology 133, 619–631 10.1053/j.gastro.2007.05.018 17681181 PMC3130597

[B35] Pitzer C. Klussmann S. Kruger C. Letellier E. Plaas C. Dittgen T. (2010). The hematopoietic factor granulocyte-colony stimulating factor improves outcome in experimental spinal cord injury. J. Neurochem. 113, 930–942 10.1111/j.1471-4159.2010.06659.x 20202082

[B36] Pitzer C. Kruger C. Plaas C. Kirsch F. Dittgen T. Muller R. (2008). Granulocyte-colony stimulating factor improves outcome in a mouse model of amyotrophic lateral sclerosis. Brain 131, 3335–3347 10.1093/brain/awn243 18835867 PMC2639207

[B37] Roberts A. W. (2005). G-CSF: a key regulator of neutrophil production, but that's not all! Growth Factors 23, 33–41 10.1080/08977190500055836 16019425

[B38] Rommel C. Bodine S. C. Clarke B. A. Rossman R. Nunez L. Stitt T. N. (2001). Mediation of IGF-1-induced skeletal myotube hypertrophy by PI(3)K/Akt/mTOR and PI(3)K/Akt/GSK3 pathways. Nat. Cell Biol. 3, 1009–1013 10.1038/ncb1101-1009 11715022

[B39] Savarese T. M. Mitchell K. Mcquain C. Campbell C. L. Guardiani R. Wuu J. (2001). Coexpression of granulocyte colony stimulating factor and its receptor in primary ovarian carcinomas. Cancer Lett. 162, 105–115 10.1016/S0304-3835(00)00623-6 11121868

[B40] Schertzer J. D. van der Poel C. Shavlakadze T. Grounds M. D. Lynch G. S. (2008). Muscle-specific overexpression of IGF-I improves E-C coupling in skeletal muscle fibers from dystrophic mdx mice. Am. J. Physiol. Cell Physiol. 294, C161–C168 10.1152/ajpcell.00399.2007 17989207

[B41] Shimoji K. Yuasa S. Onizuka T. Hattori F. Tanaka T. Hara M. (2010). G-CSF promotes the proliferation of developing cardiomyocytes *in vivo* and in derivation from ESCs and iPSCs. Cell Stem Cell 6, 227–237 10.1016/j.stem.2010.01.002 20207226

[B42] Sinclair A. M. Coxon A. McCaffery I. Kaufman S. Paweletz K. Liu L. (2010). Functional erythropoietin receptor is undetectable in endothelial, cardiac, neuronal, and renal cells. Blood 115, 4264–4272 10.1182/blood-2009-10-248666 20124513

[B43] Stitt T. N. Drujan D. Clarke B. A. Panaro F. Timofeyva Y. Kline W. O. (2004). The IGF-1/PI3K/Akt pathway prevents expression of muscle atrophy-induced ubiquitin ligases by inhibiting FOXO transcription factors. Mol. Cell 14, 395–403 10.1016/S1097-2765(04)00211-4 15125842

[B44] Stratos I. Rotter R. Eipel C. Mittlmeier T. Vollmar B. (2007). Granulocyte-colony stimulating factor enhances muscle proliferation and strength following skeletal muscle injury in rats. J. Appl. Physiol. 103, 1857–1863 10.1152/japplphysiol.00066.2007 17717125

[B45] Stupka N. Schertzer J. D. Bassel-Duby R. Olson E. N. Lynch G. S. (2008). Stimulation of calcineurin Aalpha activity attenuates muscle pathophysiology in mdx dystrophic mice. Am. J. Physiol. Regul. Integr. Comp. Physiol. 294, R983–R992 10.1152/ajpregu.00375.2007 18199592

[B46] Tachibana M. Miyakawa A. Uchida A. Murai M. Eguchi K. Nakamura K. (1997). Granulocyte colony-stimulating factor receptor expression on human transitional cell carcinoma of the bladder. Br. J. Cancer 75, 1489–1496 10.1038/bjc.1997.254 9166942 PMC2223497

[B47] Takano H. Qin Y. Hasegawa H. Ueda K. Niitsuma Y. Ohtsuka M. (2006). Effects of G-CSF on left ventricular remodeling and heart failure after acute myocardial infarction. J. Mol. Med. 84, 185–193 10.1007/s00109-005-0035-z 16418824

[B48] Takano H. Ueda K. Hasegawa H. Komuro I. (2007). G-CSF therapy for acute myocardial infarction. Trends Pharmacol. Sci. 28, 512–517 10.1016/j.tips.2007.09.002 17888521

[B49] Tanaka M. Kikuchi H. Ishizu T. Minohara M. Osoegawa M. Motomura K. (2006). Intrathecal upregulation of granulocyte colony stimulating factor and its neuroprotective actions on motor neurons in amyotrophic lateral sclerosis. J. Neuropathol. Exp. Neurol. 65, 816–825 10.1097/01.jnen.0000232025.84238.e1 16896315

[B50] Ueda K. Takano H. Hasegawa H. Niitsuma Y. Qin Y. Ohtsuka M. (2006). Granulocyte colony stimulating factor directly inhibits myocardial ischemia-reperfusion injury through Akt-endothelial NO synthase pathway. Arterioscler. Thromb. Vasc. Biol. 26, e108–e113 10.1161/01.ATV.0000219697.99134.10 16574892

[B51] Wallace M. A. Hock M. B. Hazen B. C. Kralli A. Snow R. J. Russell A. P. (2011). Striated muscle activator of Rho signalling (STARS) is a PGC-1α/oestrogen-related receptor-α target gene and is upregulated in human skeletal muscle after endurance exercise. J. Physiol. 589, 2027–2039 10.1113/jphysiol.2011.205468 21486805 PMC3090601

[B52] Walsh K. Perlman H. (1997). Cell cycle exit upon myogenic differentiation. Curr. Opin. Genet. Dev. 7, 597–602 10.1016/S0959-437X(97)80005-6 9388774

[B53] Ward A. C. Hermans M. H. Smith L. van Aesch Y. M. Schelen A. M. Antonissen C. (1999). Tyrosine-dependent and -independent mechanisms of STAT3 activation by the human granulocyte colony-stimulating factor (G-CSF) receptor are differentially utilized depending on G-CSF concentration. Blood 93, 113–124 9864153

[B54] Yaffe D. Saxel O. (1977). Serial passaging and differentiation of myogenic cells isolated from dystrophic mouse muscle. Nature 270, 725–727 10.1038/270725a0 563524

[B55] Yan B. Wei J. J. Yuan Y. Sun R. Li D. Luo J. (2013). IL-6 cooperates with G-CSF to induce protumor function of neutrophils in bone marrow by enhancing STAT3 activation. J. Immunol. 190, 5882–5893 10.4049/jimmunol.1201881 23630344

[B56] Yata K. Matchett G. A. Tsubokawa T. Tang J. Kanamaru K. Zhang J. H. (2007). Granulocyte-colony stimulating factor inhibits apoptotic neuron loss after neonatal hypoxia–ischemia in rats. Brain Res. 1145, 227–238 10.1016/j.brainres.2007.01.144 17359943 PMC1888563

