# Corrigendum: G-CSF does not influence C2C12 myogenesis despite receptor expression in healthy and dystrophic skeletal muscle

**DOI:** 10.3389/fphys.2017.00886

**Published:** 2017-10-30

**Authors:** Craig R. Wright, Erin L. Brown, Paul A. Della-Gatta, Alister C. Ward, Gordon S. Lynch, Aaron P. Russell

**Affiliations:** ^1^Centre for Physical Activity and Nutrition, School of Exercise and Nutrition Sciences, Deakin University, Burwood, VIC, Australia; ^2^Molecular and Medical Research SRC, School of Medicine, Deakin University, Waurn Ponds, VIC, Australia; ^3^Basic and Clinical Myology Laboratory, Department of Physiology, The University of Melbourne, VIC, Australia

**Keywords:** G-CSF, cytokine receptor, skeletal muscle, duchenne muscular dystrophy, *mdx*, C2C12, proliferation, differentiation

In the original article, there was a mistake in Figure 1. Identification of G-CSFR in human and rodent skeletal muscle, as published. The mistake is found in part 1A of the figure which should show that PCR product from mouse tissues (left hand image) and human tissues (right hand image). Unfortunately, when preparing the image the right hand agarose gel image that was embedded is the same as the left hand image. The corrected Figure 1 and figure legend appears below. The authors apologize for this error and state that this does not change the scientific conclusions of the article in any way.

**Figure 1:**
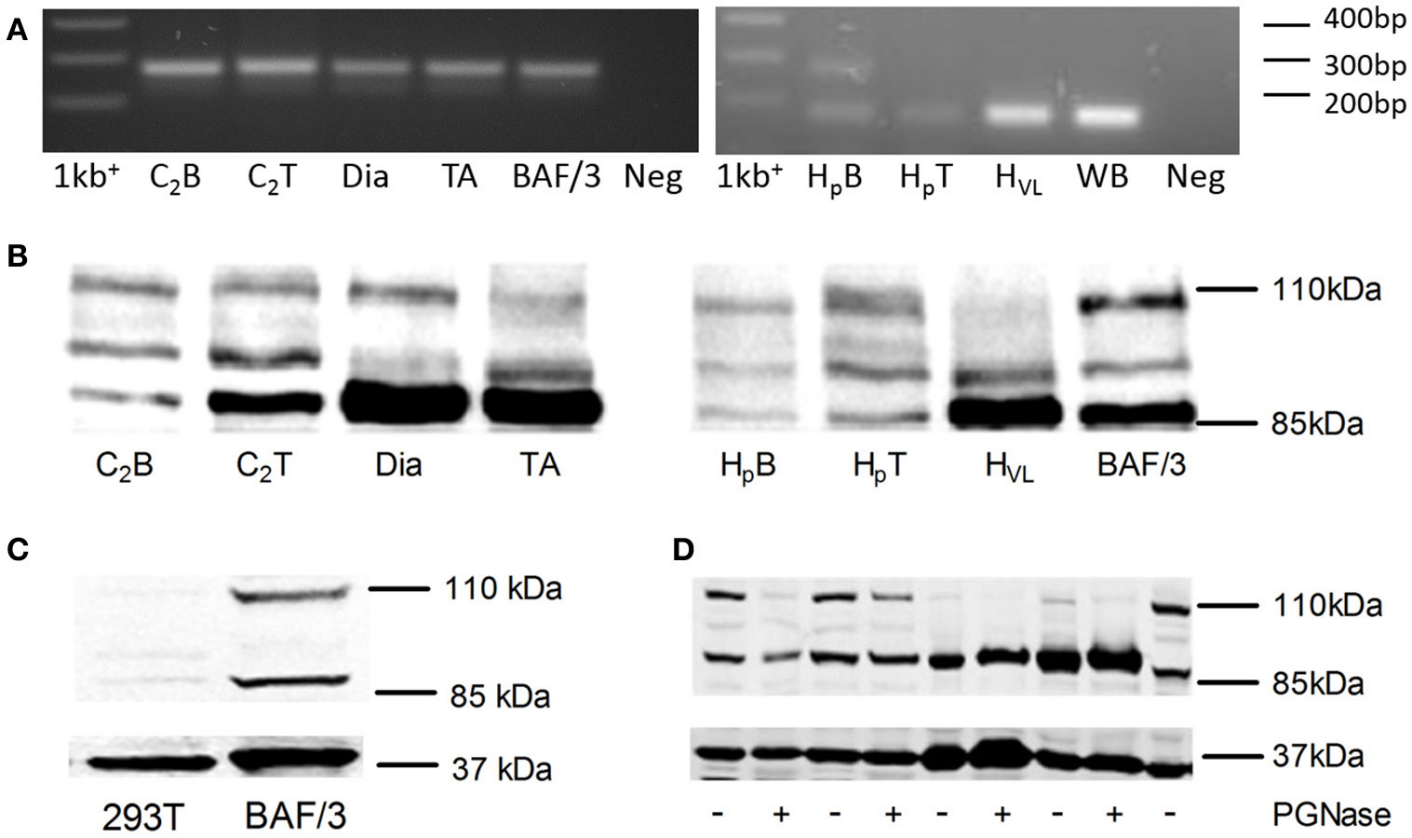
Identification of G-CSFR in rodent and human skeletal muscle. **(A)** cDNA fragment amplified during Real Time-PCR using the primers described in Table 1, separated on a 1.8% Sybr safe (Invitrogen) agarose gel and exposed to UV light. **(B)** Western blot image identifying G-CSFR in rodent and human skeletal muscle *in vitro* and *ex vivo*. **(C)** Western blot for G-CSFR in positive (BAF/3[G]) and negative (293T) control cells. **(D)** G-CSFR after deglycosylation with PGNase. C_2_B (C_2_C_12_ myoblasts), C_2_T (C_2_C_12_ myotubes), Dia (diaphragm muscle from C57BLK mice), TA (tibialis anterior muscle from C57BLK mice), H_p_B (human primary myoblasts), H_p_T (human primary myotubes) H_VL_ (human vastus lateralis muscle), BAF/3[G] (murine pro B cell line overexpressing G-CSFR), 293T (human embryonic kidney 293T cell line), 1kb^+^ [1 kb plus ladder (Invitrogen) and WB (Whole blood (human)].

## Conflict of interest statement

The authors declare that the research was conducted in the absence of any commercial or financial relationships that could be construed as a potential conflict of interest. The reviewer BGH and handling Editor declared their shared affiliation.

