# Supplementary figures and images for: G-CSF does not influence C2C12 myogenesis despite receptor expression in healthy and dystrophic skeletal muscle
Source: Front Physiol. 2014 May 1;5:170. doi: 10.3389/fphys.2014.00170 (PMC4013466; doi:10.3389/fphys.2014.00170)

24 Hrs

48 Hrs

72 Hrs

96 Hrs

Control

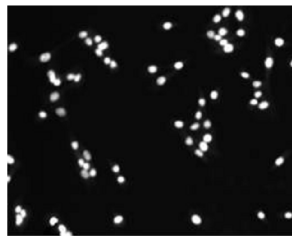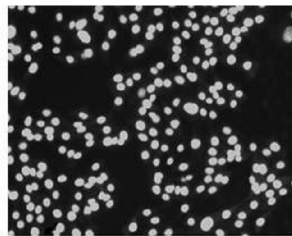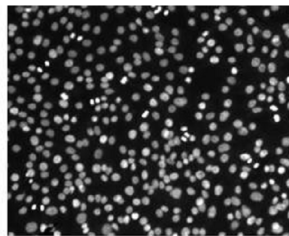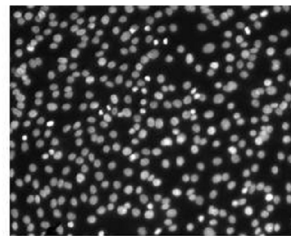

0.4 ng/ml  
G-CSF

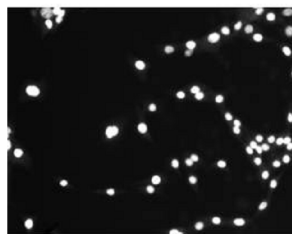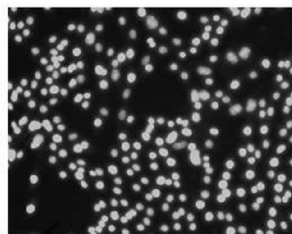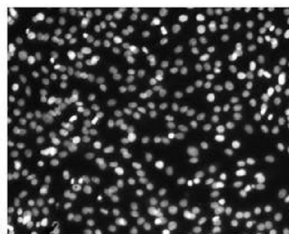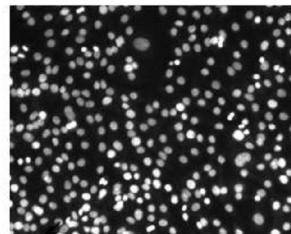

4 ng/ml  
G-CSF

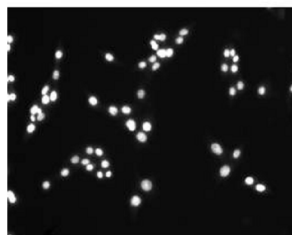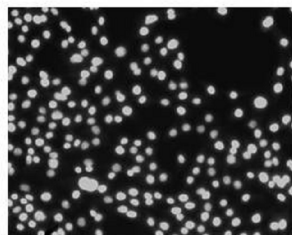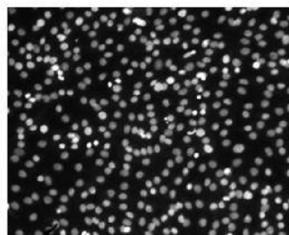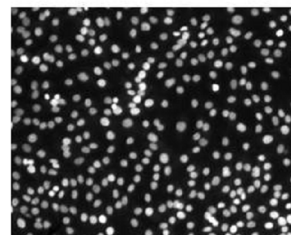

40 ng/ml  
G-CSF

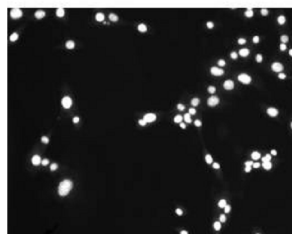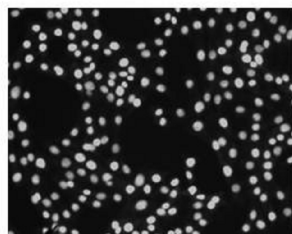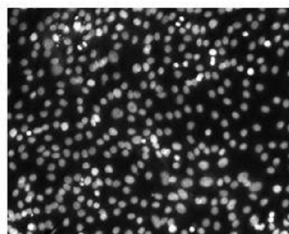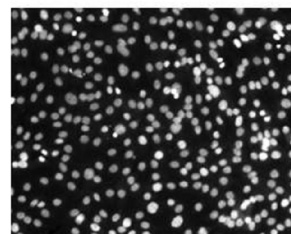

100 ng/ml  
G-CSF

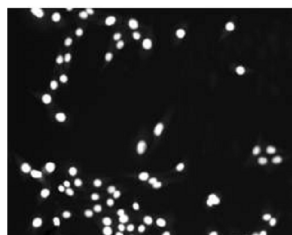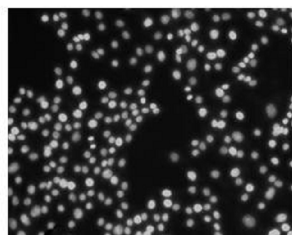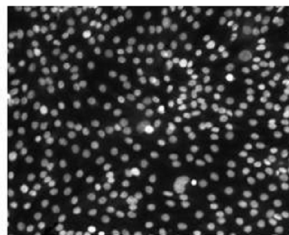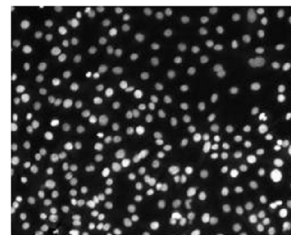

Supplement: Supplementary Figure 1 — BrDU incorporation into proliferating C2C12 myoblasts following G-CSF treatment. Proliferating C2C12 myoblasts in Growth Media (GM) containing DMEM + 10% FBS (black bars) or DMEM + 2% BSA (white bars) following 0, 0.4, 4, 40, and 100 ng/ml G-CSF treatment for (1) 24 h and (2) 48 h. Data is Mean ± SEM, n = 8, *p < 0.05 compared to GM. [file Presentation1.PDF]

A

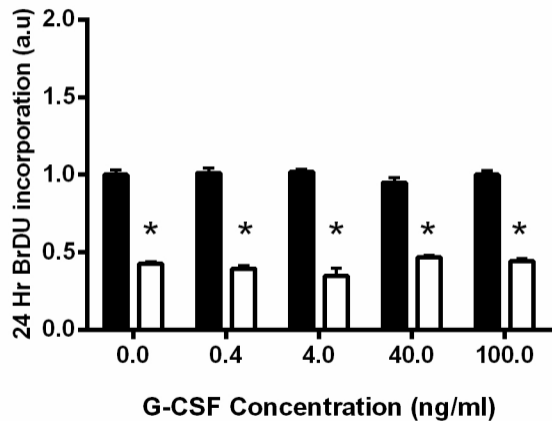

B

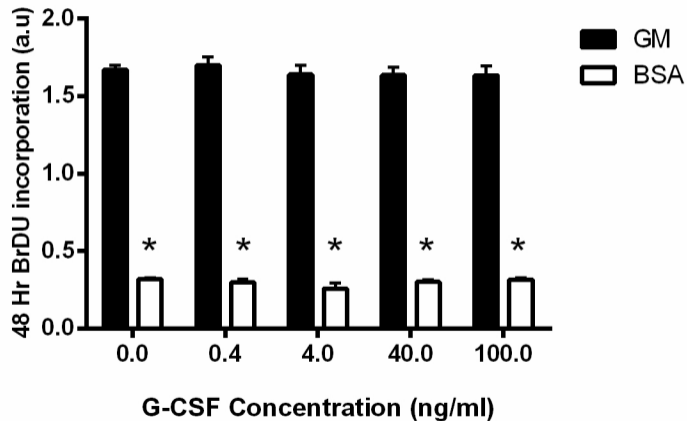

Supplement: Supplementary Figure 2 — DAPI staining of proliferating C2C12 myoblast following G-CSF stimulation grown in 10% FBS. Representative images following DAPI staining of C2C12myoblastsfollowing 24, 48, 72, and 96 h in DMEM + 10% FBS with the indicated concentration of G-CSF. A minimum of 10 images were obtained per sample (n = 3). [file Presentation2.PDF]

24 Hrs

48 Hrs

72 Hrs

96 Hrs

Control

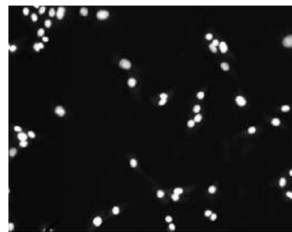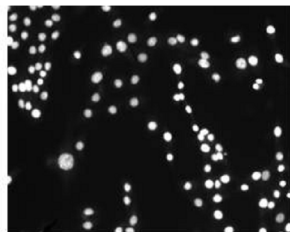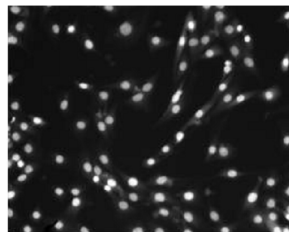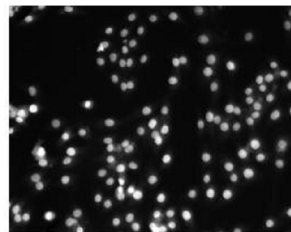0.4 ng/ml  
G-CSF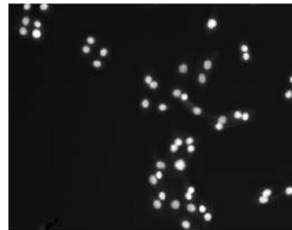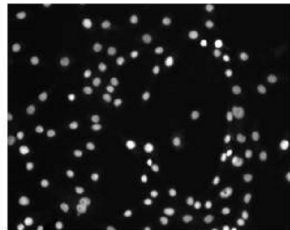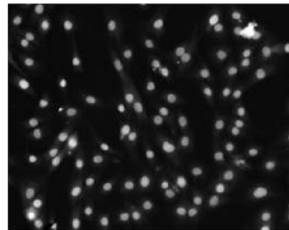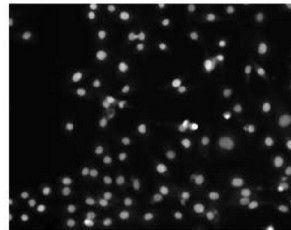4 ng/ml  
G-CSF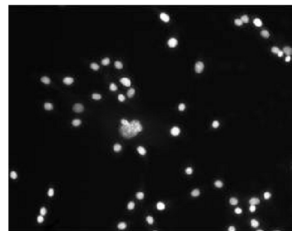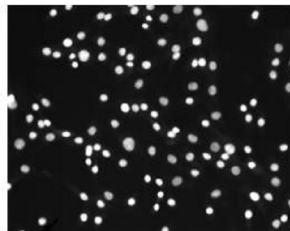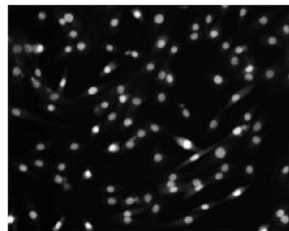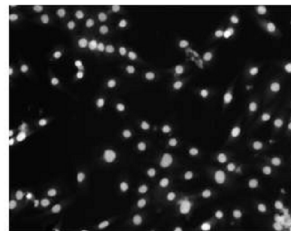40 ng/ml  
G-CSF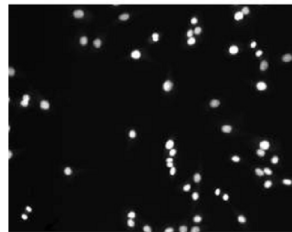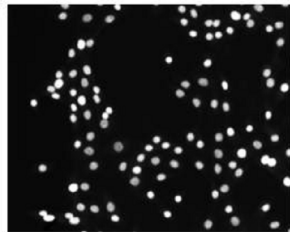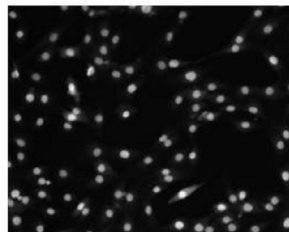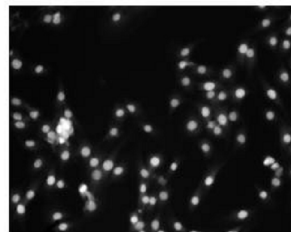100 ng/ml  
G-CSF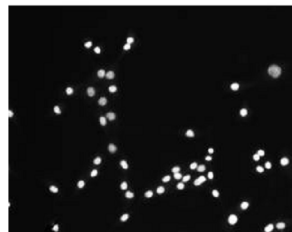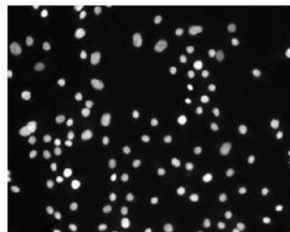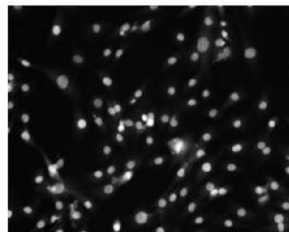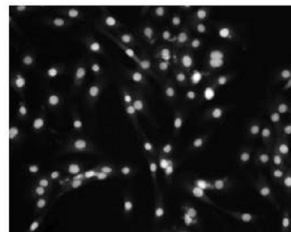

Supplement: Supplementary Figure 3 — DAPI staining of proliferating C2C12 myoblasts following G-CSF treatment with serum depletion. Representative images following DAPI staining of C2C12myoblastsfollowing 24, 48, 72, and 96 h in DMEM + 2% BSA with the indicated concentration of G-CSF. A minimum of 10 images were obtained per sample (n = 3). [file Presentation3.PDF]
