# Supplementary material for: G-CSF does not influence C2C12 myogenesis despite receptor expression in healthy and dystrophic skeletal muscle
Source: Front Physiol. 2014 May 1;5:170. doi: 10.3389/fphys.2014.00170 (PMC4013466; doi:10.3389/fphys.2014.00170)
Supplement: Supplementary file 4 [file DataSheet1.DOCX]

**Supplementary table 1 G-CSFR mRNA sequencing results.**

| Cell line | Sequencing Primer | AGRF Sequencing results |
| --- | --- | --- |
| BAF/3[G] | Murine G-CSFR  Sense (5’-3’) | NNNNNNNNGNCTTACCCTGAGGNNCCTAGATCCNTCTGACTTAAACATTTTCCTGGGCATACTTTGCTTAGTACTCTTGTCCACTACCTGTGTAGTGACCTGGCTCTGCTGCAAACGCAGAGGAAAGACTTCCTTCTGGTCAGATGTGCCAGACCCAGCCCACAGTAGCCTGAGCTCCTGGTTGCCCACCATCATGACAGAGGAAACCTTCCAGTTACCCAGCTTCTGGGACTCCAGCGTGAA |
| BAF/3[G] | Murine G-CSFR  Anti-sense (3’-5’) | NNNNNNNNNNNNNGTCNTGATGGTGNNNNNCAGGAGCTCAGGCTACTGTGGGCTGGGTCTGGCACATCTGACCAGAAGGAAGTCTTTCCTCTGCGTTTGCAGCAGAGCCAGGTCACTACACAGGTAGTGGACAAGAGTACTAAGCAAAGTATGCCCAGGAAAATGTTTAAGTCAGATGGATCTAGGGTCCTCAGGGTAAGGCCTGTACTATTGGTGGACCCTGCTCGACTGGTGGCCATGAAA |
| C_2_C_12_ myoblasts | Murine G-CSFR  Sense (5’-3’) | NNNNNNNNNNTTANCCTGAGGANCCTAGATCCNTCTGACATAAACATTTTCCTGGGCATACTTTGCTTAGTACTCTTGTCCATTACCTGTGTAATCGCCTGGCTCTGCTACAAGCACAGAGGAAAGACTTTCTTCTGGTCAAATGTGCCAGACCCAGCCCACAGTAGCCTGAGCTCCTGGTTCCCAACATCATGACAGAGGAAACCTTCCAGTTACCCAGCTTCTGGGACTCCAGCGTGAA |
| C_2_C_12_ myoblasts | Murine G-CSFR  Anti-sense (3’-5’) | NNNNNNNNNNNNNNGNCNTGATGTTGGGACCNGGAGCTCAGGCTACTGTGGGCTGGGTCTGGCACATTTGACCAGAAGAAAGTCTTTCCTCTGTGCTTGTAGCAGAGCCAGGCGATTACACAGGTAATGGACAAGAGTACTAAGCAAAGTATGCCCAGGAAAATGTTTATGTCAGATGGATCTAGGGTCCTCAGGGTAAGACCTGTACTGTTGGTGGACCCTGCTCGACTGGTGGCCANNNNN |
| C_2_C_12_ Myotubes | Murine G-CSFR  Sense (5’-3’) | NNNNNNNNGNCTTACCCTGAGGNNCCTAGATCCNTCTGACTTAAACATTTTCCTGGGCATACTTTGCTTAGTACTCTTGTCCATTACCTGTGTAATCGCCTGGCTCTGCTACAAGCACAGAGGAAAGACTTTCTTCTGGTCAAATGTGCCAGACCCAGCCCACAGTAGCCTGAGCTCCTGGTTGCCCACCATCATGACAGAGGAAACCTTCCAGTTACCCAGCTTCTGGGACTCCAGCGTGAA |
| C_2_C_12_ myotubes | Murine G-CSFR  Anti-sense (3’-5’) | NNNNNNNNNNNNNNGNCNTGATGTTGGGACCNGGAGCTCAGGCTACTGTGGGCTGGGTCTGGCACATTTGACCAGAAGAAAGTCTTTCCTCTGTGCTTGTAGCAGAGCCAGGCGATTACACAGGTAATGGACAAGAGTACTAAGCAAAGTATGCCCAGGAAAATGTTTAAGTCAGATGGATCTAGGGTCCTCAGGGTAAGGCCTGTACTATTGGTGGACCCTGCTCGACTGGTGGCCATGNNN |
